# Mitochondria-to-plasma-membrane H_2_O_2_ signaling induced by branched-chain keto acid oxidation essentially stimulates insulin secretion in pancreatic β-cells

**DOI:** 10.1016/j.redox.2026.104309

**Published:** 2026-07-16

**Authors:** Eduardo Klöppel, Hana Engstová, Pavla Duspiva, Jan Tauber, Oleksandra Mozheitova, Martin Jabůrek, Petr Ježek

**Affiliations:** Department of Mitochondrial Physiology, Institute of Physiology of the Czech Academy of Sciences, No.75, Vídeňská 1083, Prague, 14220, Czech Republic

**Keywords:** *Pancreatic β-cells*, *Branched-chain keto acids/ insulin secretion*, *Mitochondrial H*_*2*_*O*_*2*_*signaling*, *Electron-transfer flavoprotein ubiquinone oxidoreductase*

## Abstract

Oxidation (β-like) of branched-chain keto acids (BCKAs) α-ketoisocaproate (KIC), α-ketoisovalerate (KIV), and α-ketomethylvalerate (KMV), yields FADH_2_, NADH (in order of KIC < KMV < KIV), and acetyl-CoA (KIC, KMV) or succinyl-CoA (KIV, KMV). Here, we examined whether BCKA-oxidation contributes to H_2_O_2_-dependent redox signaling, studied mechanism(s) of its generation, and investigated whether such H_2_O_2_ signal is required for BCKA-stimulated insulin secretion (BCKA-SIS) in pancreatic β-cells and islets. Using Amplex UltraRed, we detected BCKA-induced H_2_O_2_ release to the exterior of INS-1E cells and pancreatic islets (PIs) upon BCKA-SIS. This H_2_O_2_ signal determined closure of ATP-sensitive K^+^ channels and enabled Ca^2+^oscillations. It was inhibited by the mitochondrial antioxidant SkQ1; partially by S1QEL, S3QEL (Complex I, III) superoxide-suppressors and by 80–90% after silencing of electron-transfer flavoprotein (ETF) ubiquinone (Q) oxidoreductase (ETFQOR). The H_2_O_2_ (redox) signal is generated *i*) due to the excessive ETFQOR QH_2_ input, which retards respiratory chain electron transport, providing surplus superoxide at Complex I site I_Q_ (i.e., reversed electron transfer, representing an effective product inhibition of the Complex I QH_2_ output) and *ii*) due to the excessive incoming QH_2_ to the Complex III site III_Qo_ (minimum for KIV). ^13^C-incorporation from U-^13^C-KIC/KIV into various metabolites confirmed β-like oxidation and characterized auxiliary reactions. A causal dependence of BCKA-SIS in PIs on H_2_O_2_ generation was found at both phases, evidenced by non-constant correlations of insulin release vs. H_2_O_2_ release rates, similarly to glucose-stimulated insulin secretion. Thus, BCKA-stimulated insulin secretion requires coordinated peri-plasma-membrane elevations of ATP/ADP and H_2_O_2_, both arising from mitochondrial BCKA β-like oxidation.

## Highlights

• Branched-chain keto acid β-like oxidation generates a mitochondrial H_2_O_2_ signal.
• Peri-plasma-membrane H_2_O_2_ and ATP elevations jointly promote insulin secretion.
• Mitochondrial origin of signal stems from its block by SkQ1 and ETFQOR silencing.
• Excessive ubiquinol input by ETFQOR creates superoxide within respiratory chain.

## Graphical abstract

**Figure:**
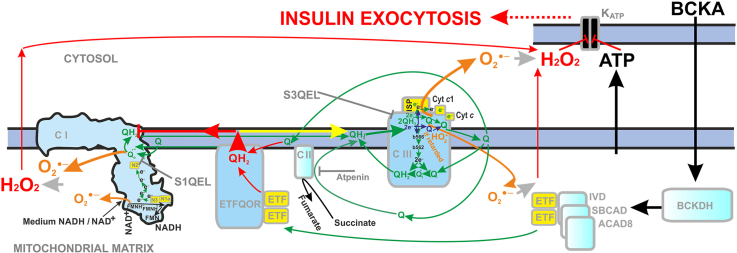

## Abbreviations

2-HG –: 2-hydroxyglutarate
ACSF3 –: acyl-CoA synthetase 3
ALDH2 –: aldehyde dehydrogenase 2
ALT1,2 –: alanine aminotransferase cytosolic, mitochondrial (glutamate-pyruvate transaminase 1,2; GPT1,2)
AST1,2 –: aspartate aminotransferase cytosolic, mitochondrial (glutamate-oxaloacetate transaminase 1,2; GOT)
AUC –: area under curve
BCAT1,2: branched-chain amino acid transferase cytosolic, mitochondrial
BCKAs –: branched-chain keto acids
BCKA-SIS –: branched-chain-keto-acid-stimulated insulin secretion
BCKDH –: branched-chain keto acid dehydrogenase
BDH –: β-hydroxybutyrate dehydrogenase (3-hydroxybutyrate dehydrogenase)
BSA –: bovine serum albumin
r-BEL –: r-bromoenol-lactone
CRAT –: carnitine-O-acetyltransferase
ETF –: electron-transfer flavoprotein
ETFQOR – electron-transfer flavoprotein: ubiquinone-oxido-reductase
FA –: fatty acid
FASIS –: fatty-acid-stimulated insulin secretion
GT1,2 –: glutaminase cytosolic, mitochondrial
GPR40 –: G-protein-coupled receptor-40
GSIS –: glucose-stimulated insulin secretion
HRP –: horseradish peroxidase
HMG –: 3-hydroxy-3-methylglutaryl
iPLA_2_γ/PNPLA8 –: mitochondrial redox-sensitive phospholipase A_2_, isoform γ/patatin-like phospholipase domain-containing protein 8
IDH2 –: isocitrated dehydrogenase 2 (NADPH dependent) mitochondrial
K_ATP_ –: ATP-sensitive K^+^-channel
KIC –: α-ketoisocaproate (2-oxoisocaproate, 4-methyl-2-oxopentanoate)
KIV –: α-ketoisovalerate (3-methyl-2-oxobutyrate, 2-keto-3-methyl-butyrate, 3-methyl-2-oxobutanoate)
KMV –: α-ketomethylvalerate (3-methyl-2-oxopentanoate, 2-keto-3-methylvalerate)
KRH –: Krebs Ringer HEPES buffer
MAGs –: monoacylglycerols
MMUT –: Methylmalonyl-CoA mutase
mt –: mitochondrial
MTBE –: methyl-*tert*-butyl ether
mt-PM redox signal –: redox signal from mitochondria to the plasma membrane
NOX4 –: NADPH oxidase, isoform 4
OAA –: oxalo acetate
OXCT1 –: 3-oxoacid CoA-transferase 1 (succinyl-CoA-transferase, SCOT1)
OXPHOS –: oxidative phosphorylation
Pcarbox –: pyruvate carboxylase
PCCB –: propionyl-CoA carboxylase
PIs –: pancreatic islets
PHGDH –: phosphoglycerate dehydrogenase
PNPLA8/iPLA_2_γ –: patatin-like phospholipase domain-containing protein 8/mitochondrial redox-sensitive phospholipase A_2_, isoform γ
QH_2_/Q –: Coenzyme Q (reduced/oxidized)
ROI –: regions of interest
ROS –: reactive oxygen species
S1QEL, S3QEL –: suppressor of Complex I site Q electron leak, suppressor of Complex III site Q electron leak
s-BEL –: s-bromoenol-lactone
S-CoA –: succinyl-CoA
SCOT1 –: succinyl-CoA-transferase (3-oxoacid CoA-transferase 1, OXCT1)
SDHD –: succinate dehydrogenase (Complex II) subunit D
SM –: Supplementary Material
TGs –: triglycerides

## 1 Introduction

**Fig. 1:**
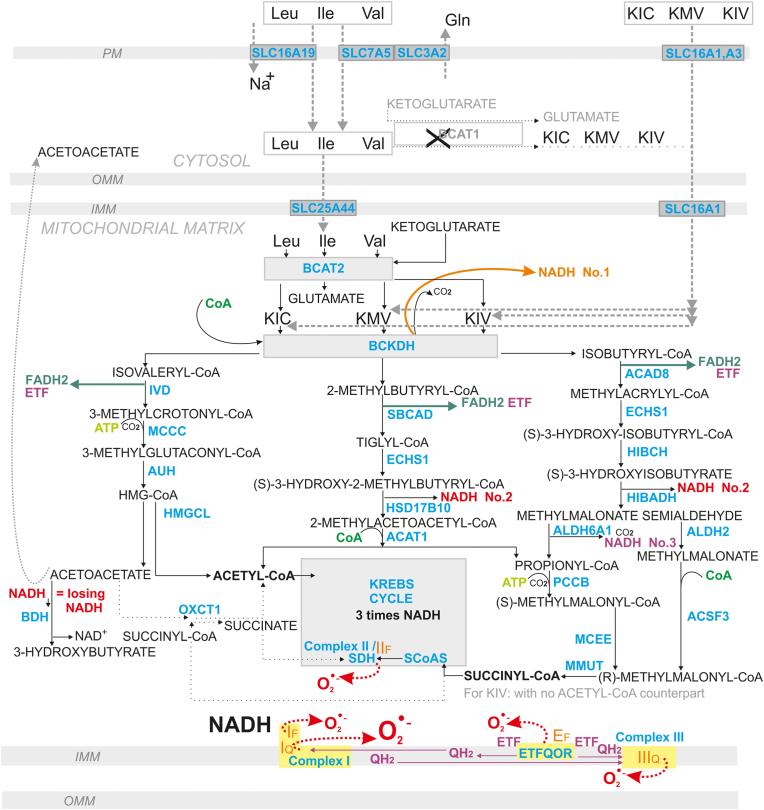
**BCKA β-like oxidation vs. possible sites of superoxide formation in pancreatic β-cells** – Metabolism of each individual BCKA is depicted with emphasis on common features (cf. e.g. Refs. [1,2]). These are following: *i*) the reaction of BCKA-dehydrogenase (BCKDH) complex, existing exclusively in mitochondria and providing decarboxylation of BCKAs with CoA as a co-substrate on its E1 and E2 subunits and at the expense of NAD^+^ on the E3 subunit to form the respective CoA derivatives and the first NADH molecule (“NADH No.1”), which potentially contributes to superoxide formation on the Complex I site I_F_ [3]. The E3 subunit re-oxidizes the reduced lipoyl sulfur of E2 using FAD^+^ as a cofactor, which becomes FADH_2_ and converts NAD ^+^ to the above-mentioned NADH. *ii*) In the 2nd-step, dehydrogenases also use FAD ^+^ as a cofactor which turns to FADH_2_, thus transferring electrons to electron transfer flavoproteins (ETFs). Next, two ETFs are required to interact with ETF-ubiquinone oxido-reductase (ETFQOR), located at the matrix surface of the inner mitochondrial membrane (IMM), which takes one electron, one at a time, from ETFs. This is coupled with the iron clusters of ETFQOR, reducing ubiquinone (coenzyme Q) to ubiquinol (QH_2_). Next, QH_2_ enters a common IMM QH_2_ pool, to which major contributors are Complex I and II; and which is consumed by so-called Q-cycle in Complex III of the respiratory chain. According to the nomenclature of M. Brand [3], sites I_F_ and I_Q_ of Complex I and III_Qo_ of Complex III are considered to form superoxide upon forward, i.e., normal electron transport within the respiratory chain, while the flavin site E_F_ of ETFQOR was found to have a minor contribution in skeletal muscle [4]. Disputes exist, whether a reverse electron transfer from the excessively accumulated succinate favors superoxide production in site I_F_ [5] or I_Q_ [3]. Excessive ETFQOR-mediated input of QH_2_ could act similarly. *iii*) Final products of KIC, KMV, and KIV β-like oxidation are acetoacetate plus acetyl-CoA, or acetyl-CoA plus succinyl-CoA, or succinyl-CoA alone, respectively. Acetyl-CoA and succinyl-CoA enter the Krebs cycle. For KMV and KIV another intermediate step by HSD17B10 and HIBADH dehydrogenases, respectively, form the second NADH (“NADH No.2”) upon β-like oxidation. For KIV, the next step catalyzed by ALDH6A1 produces even the third NADH (“NADH No.3”), however, this reaction can be bypassed by the ALDH2 plus ACSF3 reaction. The succinyl-CoA input, if excessive, might also contribute to superoxide formation within the Complex II (succinate dehydrogenase, SDH, localized at the matrix IMM side). For KIC, acetoacetate may be utilized by 3-hydroxybutyrate dehydrogenase (BDH) at the expense of NADH, which would lose one NADH molecule to lower NADH balance for KIC β-like oxidation. However, its extent depends on the overall matrix NADH/NAD^+^, on the extent of acetoacetate escape, i.e., efflux to the cytosol; and on the extent of OXCT1 reaction, which is specifically enhanced, when KIC is metabolized together with KMV or KIV. For abbreviations, see their list. Other abbreviations absent in the article text are following: ACAD8 – isobutyryl-CoA dehydrogenase; ACAT1 – acetyl-CoA acetyltransferase 1; ALDH6A1 – methylmalonate semialdehyde dehydrogenase; AUH – 3-Methylglutaconyl-CoA hydratase; ECHS1 – Short-chain enoyl-CoA hydratase; HIBADH – 3-Hydroxyisobutyrate dehydrogenase; HIBCH – 3-Hydroxyisobutyryl-CoA hydrolase; HMGCL – 3-Hydroxy-3-methylglutaryl-CoA lyase; HSD17B10 – 3-Hydroxyacyl-CoA dehydrogenase type-2, termed also 17-β-Hydroxysteroid dehydrogenase X; IVD – Isovaleryl-CoA dehydrogenase; MCCC – Methylcrotonyl-CoA carboxylase; MCEE – Methylmalonyl CoA epimerase; SBCAD – Short/branched-chain acyl-CoA dehydrogenase, termed also 2-methylbutyryl-CoA dehydrogenase.

Insulin secretion in response to an exclusively protein diet is likely ensured by branched-chain keto acids (BCKAs, synonyms: oxoacids). BCKAs are important biomolecules [1,2], having the ability to change redox homeostasis upon their oxidation. They include α-ketoisocaproate (KIC; synonyms: 2-oxoisocaproate, OIC; 4-methyl-2-oxopentanoate), α-ketoisovalerate (KIV; synonyms: 3-methyl-2-oxobutyrate, 2-keto-3-methyl-butyrate, 3-methyl-2-oxobutanoate), and α-ketomethylvalerate (KMV; synonyms: 3-methyl-2-oxopentanoate, 2-keto-3-methylvalerate). Exclusive mitochondrial (mt) pathways for so-called β-like oxidation of BCKAs are different in different tissues and each of BCKAs is metabolized with different NADH yields and different entry points into the Krebs cycle (Fig. 1 and Part I of Supplementary Material (SM); Fig. S1A and B) [1,2].

In pancreatic β-cells, being metabolites of Leu, Ile, and Val from a protein diet, BCKAs likely contribute to insulin secretion [[6], [7], [8], [9], [10], [11], [12], [13]], even in the presence of low glucose, as demonstrated for KIC [14], despite their >10-fold lower concentration relative to glucose in vivo. β-Like oxidation of KIC provides mitochondrial superoxide formation [[12], [13], [14], [15], [16]], similarly as fatty acid β-oxidation [15,17]. Moreover, due to the observed inhibition of KIC-stimulated insulin secretion by the mitochondrial antioxidant SkQ1, we concluded that the KIC-induced mitochondrial superoxide/H_2_O_2_ burst represents a H_2_O_2_ signal from mitochondria to the plasma membrane (mt-PM H_2_O_2_ signal) [14]. This is fundamentally required for the closure of ATP-sensitive K^+^ channels (K_ATP_) in concert with elevated ATP, to trigger action potential spikes on the plasma membrane of pancreatic β-cells and consequent cytosolic [Ca^2+^]_c_-oscillations, leading to the exocytosis of insulin granule vesicles [[12], [13], [14]]. The ATP elevation should be supplied from the BCKA β-like oxidation followed by oxidative phosphorylation (OXPHOS).

Unfortunately, details of where and how excessive superoxide is formed are not known for BCKA β-like oxidation, similarly as no detailed knowledge exists for fatty acid (FA) β-oxidation in any tissues, despite previous attempts [3,4,[17], [18], [19], [20], [21]]. For KIC, several processes may contribute to mitochondrial superoxide formation. These include the increased entry of acetyl-CoA into the Krebs cycle (Fig. 1), the increasing load of NADH toward the Complex I of the mitochondrial respiratory chain; and an additional input of electrons from FADH_2_ (reactions ultimately following BCKA dehydrogenase; Fig. 1) into the respiratory chain via the electron-transfer flavoprotein (ETF) ubiquinone (Q) oxidoreductase (ETFQOR). All these could be potential sources of the increasing superoxide formation in mitochondria during the onset of BCKA β-like oxidation. Similar mechanisms may operate for KIV, for which besides NADH and ETF inputs, the increased entry of succinyl-CoA may contribute; as well as for KMV, for which both acetyl-CoA plus succinyl-CoA may participate. Surplus fluxes of succinyl-CoA, converted to a higher succinate load, might also partially induce superoxide formation [5] at Complex I site I_Q_ or at Complex II [22] or Complex III. Nevertheless, any precise contribution of each site to superoxide formation upon β-like oxidation is not known.

BCKA metabolic pathways can proceed superimposed on metabolism of low glucose in pancreatic β-cells. The glucose metabolism alone exhibits a theoretical steady-state yield of 3 NADH per Krebs cycle turn, if no pyruvate-based redox shuttles operate [23] under such “resting” conditions (meaning no insulin release) [14,24]. The superimposed onset of KIC, KMV, and KIV oxidation to this basal metabolism might theoretically bring another 1, 2, and 3 NADH molecules per cycle, respectively (i.e., oxidation of a single BCKA molecule; Fig. 1). However, theoretical predictions, based on knowledge gathered from different tissues (e.g. Ref. [25]), may not be fulfilled for pancreatic β-cell β-like oxidation, which may also possess different end-point reactions. These reactions concern with acetoacetate utilization for KIC by β-hydroxybutyrate dehydrogenase (BDH; 3-hydroxybutyrate dehydrogenase); utilization of methylmalonate semialdehyde for KIV by alternative aldehyde dehydrogenase 2 (ALDH2) plus acyl-CoA synthetase 3 (ACSF3); and for all BCKAs the extent may differ for the 3-oxoacid CoA-transferase 1 reaction (OXCT1, termed also succinyl-CoA-transferase, SCOT1; consuming succinyl-CoA and acetoacetate) (Fig. 1; Fig. S1). These additional reactions may alter the NADH yield as well as superoxide formation. Hence, it is necessary to determine their extent by metabolomics or silencing studies for oxidation of each individual BCKA or their combinations.

The excessive NADH formation is expected to cause a sufficient mt matrix substrate (NADH) pressure (defined here as the [NADH]_mt_/[NAD^+^]_mt_ ratio) to induce surplus superoxide formation at the Complex I site I_F_ [3]. Since rather specific conditions for superoxide formation within the SDH/Complex II were reported [22], predictions are ambiguous whether also the excessive succinyl-CoA leads to superoxide formation in conditions of otherwise forward electron transport within the Complex I. Finally, the role of ETFQOR enzyme in redox and Coenzyme Q (QH_2_/Q) homeostasis should be elucidated with respect to superoxide formation [3]. Either a certain internal retardation of electron transfer within this enzyme may produce superoxide (e.g. at site E_F_ according to Ref. [3]); or the additional QH_2_ input into the respiratory chain may provide an effective retardation of original electron transport, otherwise ongoing in the absence of such a QH_2_ input. Note that any effective electron transfer retardation leads to superoxide formation within the respiratory chain.

We now demonstrate that each BCKA stimulates insulin secretion in a manner dependent on the mt-PM H_2_O_2_ signal, ensured by H_2_O_2_ diffusion from mitochondria. We have quantified this H_2_O_2_ release, revealed certain details of the underlying metabolic pathways, and identified candidate sites of superoxide formation during BCKA oxidation in pancreatic β-cells. With isolated pancreatic islets, we characterized BCKAs as true insulin secretagogues and demonstrated the strict dependency of insulin release rates on the rates of H_2_O_2_ efflux for both phases of insulin secretion.

## 2 Materials and methods

### 2.1 Experiments with INS-1E cells

Rat insulinoma INS-1E cells were purchased from AddexBio (San Diego, CA; C0018009) and were cultured with 11 mM glucose in RPMI 1640 medium with l-glutamine, supplemented with 10 mM HEPES, 1 mM pyruvate, 5% (v/v) fetal calf serum, 50 μmol/l mercaptoethanol, 50 IU/ml penicillin, and 50 μg/ml streptomycin. Cells were seeded at 0.2·10^6^ cells/well in poly-l-lysine-coated 12-well plates one day before transfections; three days before experiments (for siRNA silencing see SM). Cells were pre-incubated for 60 min with 3 mM glucose in KRH with 0.1% BSA. Insulin release was assayed using a Rat Insulin ELISA kit (U-E type, Shibayagi Co., Shibukawa, Japan) [14]. GCaMP6 assays for Ca^2+^ oscillations in INS-1E cells were performed after transfections with purchased plasmids [17,24]. Silencer Select technology (ThermoFisher Scientific, #4390771) was chosen with following IDs: BDH1 - s138562, OXCT1 - s234928, BCKDHA - s129255. For ETFDH silencing Mission siRNAs (Merc, SASI_Rn01_00096001, SASI_Rn01_00096002) were used as an equimolar mixture. For scrambled (Scrl) samples, Mission siRNA Universal Negative Control was used (Merck, SIC001). siRNA transfection complexes were prepared using GenMute™ transfection reagent.

Fluorometric Amplex UltraRed (plus horseradish peroxidase, HRP) monitoring was performed using 10^6^ trypsinized cells in a cuvette of a Shimadzu RF5301 PC at 37 °C, using 572 nm excitation and 580 nm emission [17]. Thallium assays of ATP-sensitive K^+^ channel closure were done as previously described, taking fluxes sensitive to 1 μΜ glibenclamide [14,17]. When required, 60 min preincubation with 10 μM S1QEL, 10 μM S3QEL, or both these suppressors (10 μM, each), was performed. Their stock solutions were in ethanol and were carefully sonicated prior to their use, to avoid precipitates. Alternatively, 10 min preincubations with 10 or 100 nM SkQ1 were made. The SkQ1 stock solution was also ethanolic. Confocal microscopy using a Leica TCS SP8 was employed for the MitoSOX Red monitoring (Thermo Fisher; 4 μM, 15 min preincubated with cells) of in situ superoxide surplus release to the mitochondrial matrix by using 514 nm excitation and 610–679 nm emission. Rates, that is, integral fluorescence intensity increases (*J*^*S*^_m_) with time, were derived in the region of interest, which was the mitochondrial network [23].

### 2.2 Assays of ^13^C-incorporation

Assays of ^13^C-incorporation were performed similarly to U–^13^C-palmitic acid [17] or U–^13^C-leucine [25] assays. Briefly, U–^13^C-α-ketoisocaproate (U-^13^C-KIC) and U–^13^C-α-ketoisovalerate (U-^13^C-KIV) were purchased from Cambridge Isotope Laboratories, Inc. (Tewksbury, MA, USA). INS-1E cells were incubated for 1 h in KRH with 3 mM glucose, 2 mM glutamine, 1 mM l-carnitine, 0.1% BSA, and 7.5 mM U-^13^C-KIC or U-^13^C-KIV. Using MS, M+5 carnitines for U-^13^C-KIC and M+4 carnitines plus M+3 propionyl carnitine for U-^13^C-KIV, were analyzed as described elsewhere, being reaction products of carnitine-O-acetyltransferase (CRAT) [26], converting the respective R–CoAs of BCKA β-like oxidation. Thus ^13^C-carnitines were used as surrogates for the corresponding CoAs. In addition, for U-^13^C-KIC, M+2 acetyl-carnitine was surveyed in parallel with directly detected M+2 acetyl-CoA and M+2 Krebs cycle metabolites (Fig. S1A and B). For U-^13^C-KIV, analogous M+4 and M+3 metabolites were surveyed.

### 2.3 Metabolomics for INS-1E cells

Metabolomics was performed in the Laboratory of Metabolomics (Institute of Physiology of the Czech Academy of Sciences; see https://metabolomics.fgu.cas.cz/index.html; cf. also Ref. [27]). Bi-phase extraction method using methanol, methyl-*tert*-butyl ether (MTBE), and water provided polar metabolites (polar metabolomics) and the other with complex lipids (aka “lipidomics”). Thus, 0.4 mg of the cellular pellet was homogenized (1.5 min) with 275 μl of methanol and 275 μl of 10% methanol using a grinder. Subsequently, 1 ml of MTBE was added, and the tubes were shaken for 1 min and centrifuged at 16,000× g for 5 min at 4 °C. Next, 500 μl of the upper organic phase was collected and evaporated, why aqueous (polar) phase was saved. The dry extracts were then re-suspended using methanol containing 12- [[(cyclohexylamino) carbonyl]amino]-dodecanoic acid internal standard, shaken for 30 s, centrifuged at 16,000 × g for 5 min at 4 °C, and used for LC-MS “lipidomics” analysis.

During untargeted analyses, all detectable compounds were acquired using validated LC-MS methods (LIMeX workflow, using over 60 different internal standards). A high-resolution mass spectrometer Thermo Q Exactive Plus coupled with a liquid chromatograph (Thermo Vanquish) was used, operating in negative electrospray ionization mode to collect full scan MS1 data as well as data-dependent MS/MS spectra for all samples. While MS1 data were used for quantification, MS/MS spectra were used for compound annotation using MS/MS library search or for compound confirmation. The samples were randomized across the platform run.

The LC-MS instrumental files generated during polar metabolomic analysis were processed using MS-DIAL 4.92 software, linked to commercial MS/MS spectral libraries (NIST20, MassBank, MoNA). All compounds were quantified as peak heights and reported in “arbitrary units”. These values were used as inputs for one-dimensional or multidimensional statistical methods (e.g., Student's T-test, fold-change, heatmap, principal component analysis, partial least squares-discriminant analysis). 2-hydroxyglutarate (2HG) was not distinguished for enantiomers [28]. For lipidomics see SM, Part II.6.

Data were then normalized using locally estimated scatterplot smoothing (LOESS) with QC samples injected between 10 actual study samples to remove instrumental drifts and then normalized to the respective total ion count (TIC) before subsequent statistical analysis. The analyzed data were plotted as Volcano plots for individual polar compounds, lipid species or groups as the sum of different species of each polar or lipid category.

### 2.4 Pancreatic islet perifusion and assays

Murine pancreatic islets (PIs) isolation and perifusion were performed as described elsewhere [14,17,23] from wild-type C57Bl6/N mice of mixed sex [17]. Typically, 100–200 isolated PIs were immobilized with Bio-Gel P4 (Bio-Rad) and washed for 60 min with a continual flow of glucose-free Krebs-Ringer HEPES (KRH) buffer (135 mM NaCl, 3.6 mM KCl, 10 mM HEPES, 0.5 mM MgCl_2_, 1.5 mM CaCl_2_, 0.5 NaH_2_PO_4_, 2 mM l-glutamine, 0.2% bovine serum albumin, BSA, pH 7.4. Perifusates were collected at a flow rate of 0.5 ± 0.1 ml/min. Insulin was quantified using an HTRF Insulin Ultra-Sensitive Detection kit (Revvity Health Sciences Inc., Waltham, MA, USA). Alternatively, 10 μM Amplex UltraRed and 5 IU/ml horseradish peroxidase (HRP) were added to the perifusate after its collection from the column containing the immobilized islets [17]. Perifusion with SkQ1 was performed for 10 min prior to BCKA addition and given SkQ1 concentration was also present during the whole time-course. The latter was also done for S1QEL and S3QEL.

PI respiration was assayed using Agilent Seahorse XF Analyzer with 50 islets per well assay medium containing 5.5 mM glucose [17]. The measured respiration rates *J* were corrected for non-mitochondrial respiration determined in the presence of 5 μM rotenone plus 5 μM antimycin A. Maximum rates were estimated in the presence of 1 μM FCCP.

### 2.5 GCaMP6 assay for Ca^2+^ oscillations in pancreatic islets

Experiments were approved (Institute of Molecular Genetics committee), complying with the 2010/63/EU directive, NIH Publication No.85-23 (revised 1996) and the ARRIVE guidelines. Mice expressing GCaMP6s (slow) fluorescent Ca^2+^indicator selectively in pancreatic β-cells were created by crossing mice overexpressing GCaMP6s (i.e., mice B6. Cg-Igs7tm162.1 (tetO-GCaMP6s,CAG-tTA2)Hze/J; RRID:IMSR_JAX:031562; purchased from Jackson Laboratory, Bar Harbor, MN, USA) with B6. Cg-Tg (Ins2-Cre)25Mgn/J mice (RRID:IMSR_JAX:003573; Jackson Laboratory) analogously as described elsewhere [14]. Tail genotyping selected congenic B6. Cg-Igs7tm162.1 (tetO-GCaMP6s,CAG-tTA2)Hze Tg (Ins2-Cre)25Mgn/J mice (GCaMP6sβoe mice). Genotyping used Standard PCR Assay, Protocol 32935 (Jackson Laboratory) for GCaMP6s plus Standard PCR Assay, Protocol 22392 for Cre. The GCaMP6s fluorescence was found already in heterozygotes, which were employed for experiments. Their islets exhibited GCaMP6s emission in <50% of β-cells (SM Part II.3 Fig. S3).

PIs of ∼3-month-old GCaMP6sβoe heterozygous mice of mixed sex were isolated as described above and before each experiment, PIs were preincubated overnight with 5.5 mM glucose in the same KRH buffer with 0.1% BSA, in which monitoring was conducted. A Leica TCS SP8 confocal microscope was employed for the time-lapsed recording of integral fluorescence intensity within the individual cells ROI with excitation at 480 and emission at 510 nm. For every second, integral fluorescence intensities *F* [Ca]_c_ (t_i_) were collected from a widefield image of each responding islet cell, and data were plotted as a time course. Typically, 10 min recordings were set prior to BCKA addition at 5.5 mM glucose, followed by 40 min recordings with each of KIC, KIV, KMV; with or without the studied agents. Alternatively, increasing levels of glucose above 5.5 mM were tested. In some runs, 30 mM KCl was added at the end. The numerical derivative was calculated to identify peaks of oscillations, so that a peak at time *t*_i_ occurred for the derivative of *F* [Ca]_c_ (*t*_i_) being zero at time *t*_i_. Peaks of oscillations in each trace were sorted by intensities into ranges scaled by 1/10 of maximum intensity (deciles or 10-percentiles) and histograms were plotted for each recording as described elsewhere [17,24].

### 2.6 Statistical analysis

Biological (*N*; number of mice, PI isolations, cell passages; or for GCaMP6 confocal microscopic assay, the number of single-cell confocal images) and experimental (*n*) replicates are listed. One-way ANOVA (T-test for two quantities) was used with pairwise multiple comparisons (Tukey's test) on the pre-validated data using SigmaStat 3.1 (Systat-Software, San Jose, CA). When suitable, two-way factorial ANOVA was utilized followed by Holm-Sidak post hoc tests (Part II.2 SM).

## 3 Results

### 3.1 β-like oxidation of branched-chain keto acids promotes H_2_O_2_ diffusion to the exterior of pancreatic β-cells

**Fig. 2:**
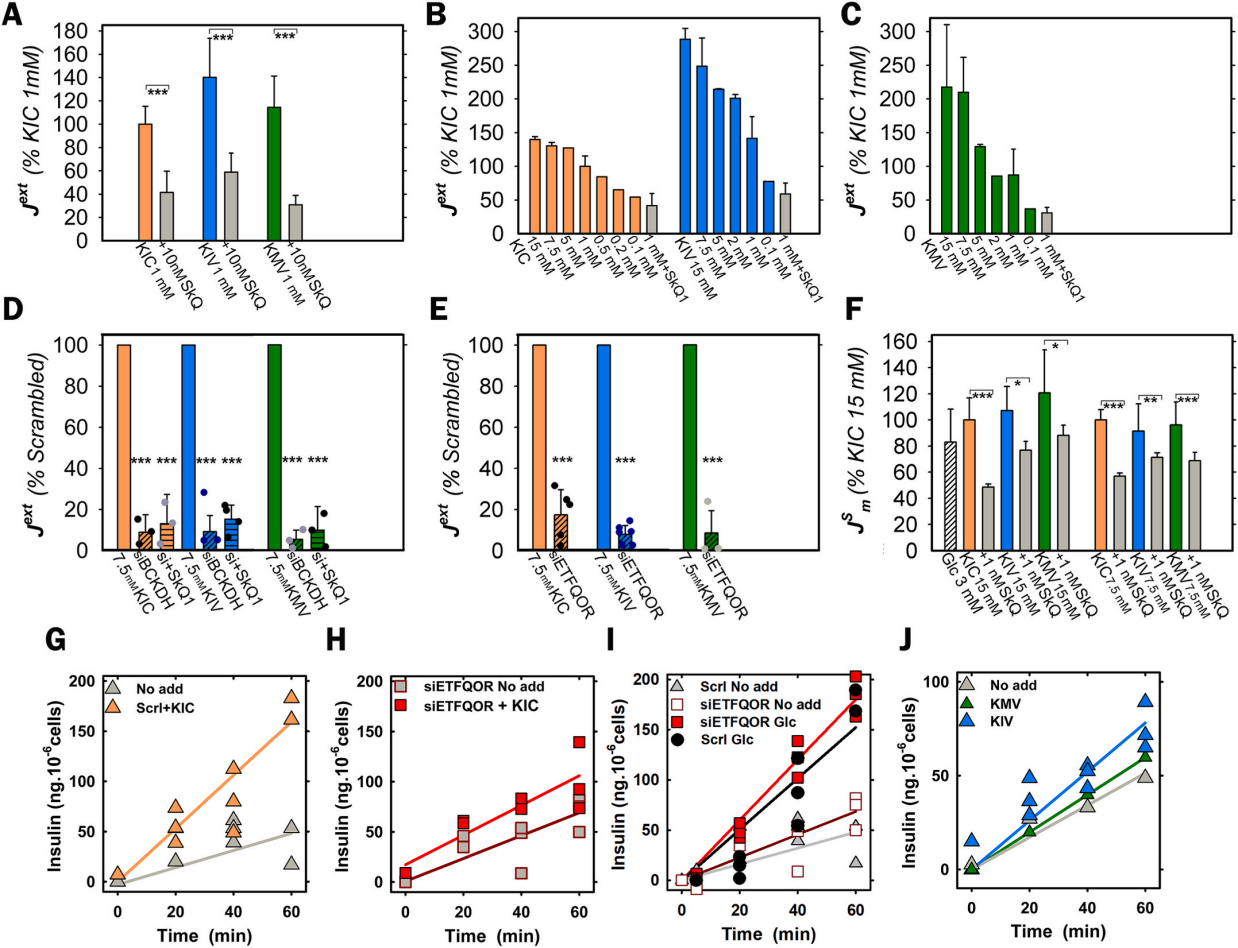
**Extracellular H_2_O_2_ release, superoxide release to the mitochondrial matrix, and insulin secretion upon β-like oxidation of BCKAs** – **A**–**E**) **Extracellular H_2_O_2_ release,** monitored with Amplex UltraRed plus HRP in INS-1E cells in the assay medium with 2 mM glutamine: Inhibition with 10 nM SkQ1 preincubated with cells for 10 min (**A**) and dose-responses (**B**,**C**) for the relative initial rates of the extracellular H_2_O_2_ release (*J*^ext^) after addition of the indicated doses of KIC (*orange*), KIV (*blue*) and KMV (*green*). **D**,**E**) Remaining activity in % of *J*^ext^ in control cells (treated with scrambled siRNA) after the silencing of BCKDH (siBCKDH, **D**) and ETFQOR (siETFQOR, **E**). For reduced transcript, protein, and description of siRNA treatment see SM, Part II.1. Rates *J*^ext0^, estimated when no BCKA was added, were subtracted from each measured rates and the resulting *J*^ext^ were normalized either to those for 1 mM KIC (**A**–**C**) or transfections with scrambled siRNA (**D**,**E**). Data was collected from *N* biological replicates (cell passages) and *n* time courses as follows: **A**–**C**) *N* = 2, *n* = 3 for KIC 15 mM; *N* = 3, *n* = 3 for KIC 7.5 mM; *N* = 10, *n* = 15 for KIC 1 mM; and *N* = 4, *n* = 8 for KIC 1 mM plus 10 nM SkQ1; similarly, *N* = 2, *n* = 3 for KIV 15 mM; *N* = 3, *n* = 7 for KIV 7.5 mM; *N* = 5, *n* = 11 for KIV 1 mM; and *N* = 5, *n* = 7 for KIV 1 mM plus 10 nM SkQ1; and *N* = 3, *n* = 3 for KIV 15 mM; *N* = 3, *n* = 4 for KIV 7.5 mM; *N* = 7, *n* = 12 for KIV 1 mM; and *N* = 5, *n* = 6 for KIV 1 mM plus 10 nM SkQ1. Scrambled siRNA was assayed *N* = 3, *n* = 3 for BCKDH siRNA treatment (*N* = 3, *n* = 3) for each BCKA. After ETFQOR siRNA treatment (*N* = 3; and pairwise treatment with scrambled siRNA) *n* = 5, *n* = 6 and *n* = 3 for KIC, KIV and KMV, respectively; ∗∗∗*P* < 0.001; ∗∗*P* < 0.05; ∗*P* < 0.1. **F**) **Superoxide release rates to the mitochondrial matrix (*J*_m_) monitored using MitoSOX Red** in INS-1E cells under a confocal microscope by the established method [23]. *N* = 3, cell passages; *n* = 6, *n* = 12, *n* = 14 and *n* = 12 for glucose, KIC, KIV and KMV, respectively. Inhibition with 1 nM SkQ1 is illustrated (*n* = 5 for each secretagogue). **G**–**J**) **Insulin secretion in INS-1E cells**. Time course of accumulated insulin after addition of 15 mM KIC (**G**,**H**), 15 mM KIV (**J**), 15 mM KMV (**J**) or 25 mM glucose (**I**) in control INS-1E cells transfected with scrambled siRNA or siRNA for ETFQOR, as indicated. For reduced transcript, protein, and description of siRNA treatment see SM. Insulin release rates were quantified after linearization of time-course of accumulated insulin and subtraction of analogous rates evaluated without BCKAs. Estimated from three assays (*N* = 3, cell passages), average differential relative rates in % of KIC insulin secretion rates were: 5.9% for KIC and siRNA ETFQOR, 25%–95% for KIV and 8%–30% for racemic KMV. Insulin secretion rates with 25 mM glucose (GSIS) amounted to 102% after siRNA ETFQOR transfection. Alternatively, these data were analyzed using two-way ANOVA, see SM, Part II.2.

We supplied individually 1 mM ketoisocaproate (KIC), ketoisovalerate (KIV), and ketomethylvalerate (KMV) to insulinoma INS-1E cells in KRH medium with 3 mM glucose plus 2 mM glutamine and externally monitored H_2_O_2_ using the Amplex UltraRed plus HRP assay [17]. The observed initial H_2_O_2_ release rates to the cell exterior (*J*^ext^) were higher for KIV vs. KIC, whereas for racemic KMV exceeded the initial *J*^ext^ rates with KIC at doses >2 mM (Fig. 2A–C). Background rates without BCKAs were subtracted for these comparisons. A similar order was found for saturated *J*_sat_^ext^ rates (SM Part II.1, Fig. S2A–C). At 1 mM BCKAs, such H_2_O_2_ release was inhibited by mitochondria-targeted antioxidant SkQ1 (by 60–70% at 10 nM; Fig. 2A–C); saturated rates were inhibited by 75–80% (Fig. S2A–C).

Silencing of BCKA dehydrogenase (BCKDH; for protein reduction see Fig. S2G and J) strongly inhibited the H_2_O_2_ release rates *J*^ext^ by ∼90%, independently of SkQ1 (Fig. 2D). Thus, the shut-down of β-like oxidation significantly blocked the H_2_O_2_ release to the cell exterior. Up to 80–90% inhibition of the initial *J*^ext^ rates for 7.5 mM BCKAs was also observed when we silenced ETF:ubiquinone-oxidoreductase (ETFQOR; ETF is the electron-transfer flavoprotein) (Fig. 2E; for protein reduction see Fig. S2H and K). Since at 1 mM BCKAs the initial acceleration was slower, the inhibitory ability of ETFQOR siRNA was apparently lower (up to 70%; Fig. S2D; for saturated *J*_sat_^ext^ rates see Fig. S2E and F). Thus, the H_2_O_2_ release to the cell exterior was absent or minimum upon interruption of the electron transfer from FADH_2_ into the QH_2_ pool of the respiratory chain. The ETFQOR deficiency had a more intensive effect than SkQ1, acting at sites I_Q_ and III_Qo_ [29]. Collectively these data demonstrate the existence of H_2_O_2_ diffusion from mitochondria to the plasma membrane (representing a H_2_O_2_ signal), induced by BCKA β-like oxidation. Moreover, we identified ETFQOR as the major input resulting in the elevated superoxide/H_2_O_2_ formation.

Rates for the superoxide release into the mitochondrial matrix (*J*^*S*^_m_), monitored using MitoSOX Red [23], were also inhibited by 1 nM SkQ1, but to a lesser extent (Fig. 2F). The highest *J*^*S*^_m_ rates among 15 mM BCKAs were found for KMV, while for KIV were higher than for KIC. At 7.5 mM BCKAs, *J*^*S*^_m_ rates for KIC were insignificantly different from those of KMV and KIV. When the SkQ1-sensitive *J*^*S*^_m_ rates were considered, they were higher for KIC than for KIV and KMV, reflecting different pattern of superoxide formation sites (Fig. S2I). Overall, we documented the existence of the increased superoxide formation in the mt matrix due to β-like oxidation of BCKAs. Elevated superoxide after dismutation to H_2_O_2_ can diffuse as H_2_O_2_ and reach the plasma-membrane. This is regarded as mt-PM H_2_O_2_ signaling. Its existence is inferred from its matrix (Fig. 2F) and exterior monitoring (Fig. 2A–E).

### 3.2 Branched-chain-keto-acid-stimulated insulin secretion (BCKA-SIS) in INS-1E cells

Next, we screened whether the observed H_2_O_2_ diffusion determines the BCKA-stimulated insulin secretion (BCKA-SIS) in INS-1E cells, assayed in KRH medium with 2 mM glutamine. Insulin release rates were taken as slopes of linearized time courses for accumulated insulin at each time point. The rates for basal secretion at 3 mM glucose were subtracted. The resulting differential insulin release rates increased in order of KIC > KIV > KMV, i.e., reciprocally to the order of *J*^*S*^_m_ and *J*^ext^ rates (all at 15 mM; Fig. 2G–J). BCKA-SIS with 15 mM KIC exhibited similar rates to GSIS at 25 mM glucose (Fig. 2G vs. 2I). At 7.5 mM KIC, average insulin release rates accounted for 38%–65% of those found for GSIS; rates at 7.5 mM KIV and KMV amounted 15%–24% of average GSIS rates. Inhibition of KIC-stimulated insulin secretion by 10 nM SkQ1 has been reported elsewhere [14].

As expected, insulin release stimulated by glucose (GSIS) was not affected by ETFQOR silencing (Fig. 2I; SM Part II.2), since the essential H_2_O_2_ signal for GSIS is provided by cytosolic NOX4 [14]. For KIC, we demonstrated that the ETFQOR silencing largely abolished the KIC-induced component of insulin secretion. This conclusion was deduced either from comparison of average differential insulin release rates (Fig. 2H) or from two-way multifactorial ANOVA analysis of rates taken from individual time points (SM Part II.2).

**Fig. 3:**
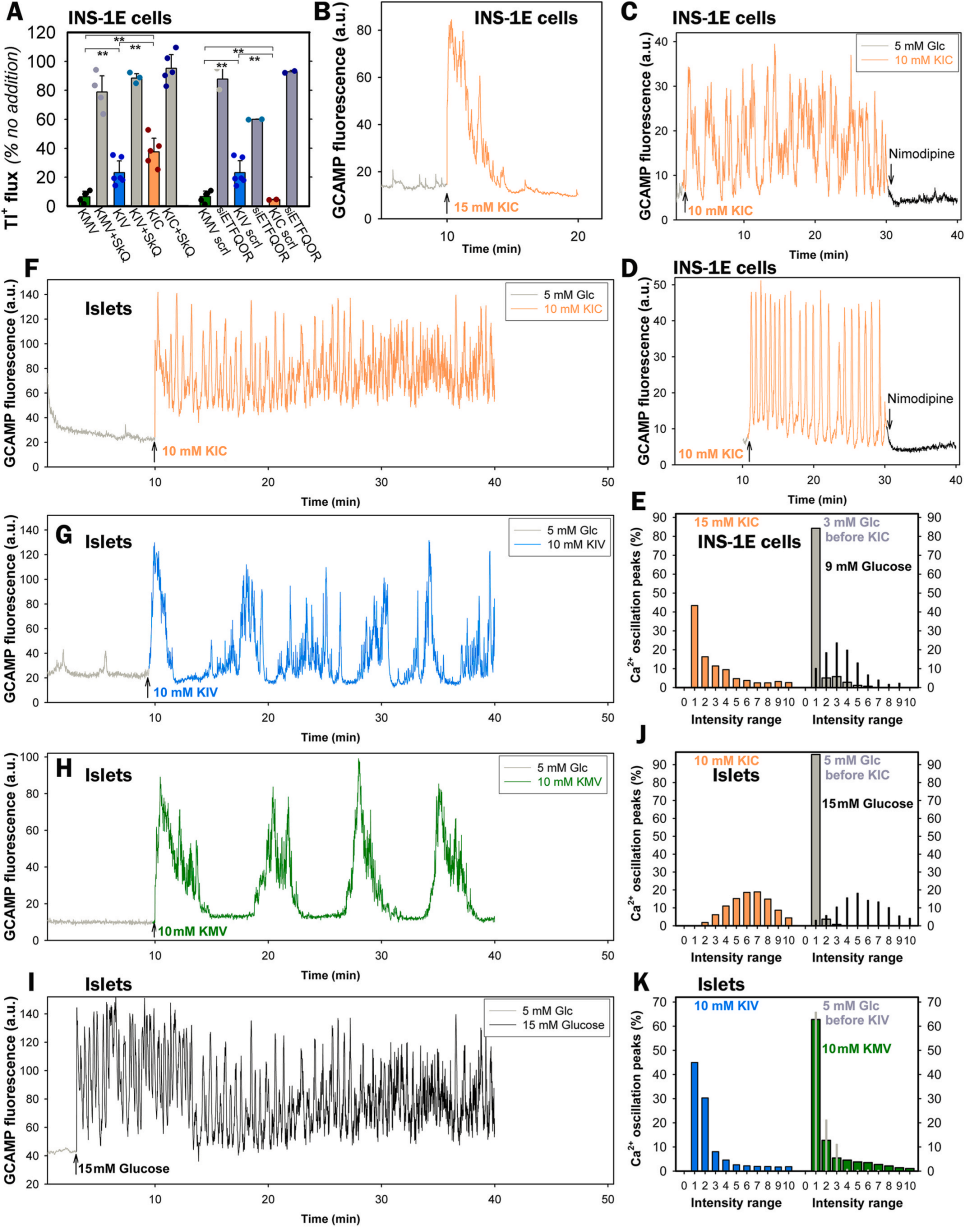
**A**) **Closure of ATP-sensitive K^+^ channels** (K_ATP_) within their entire population in INS-1E cells, induced with 15 mM BCKAs (to test SkQ1 effects) and 1 mM BCKAs (to test ETFQOR silencing). The closure state was quantified from the glibenclamide-sensitive Tl^+^ efflux (1 μΜ glibenclamide) [14,17] in the absence of presence of 10 nM SkQ1 relatively to no additions; and in ETFQOR-silenced cells, relative to those cells transfected with scrambled siRNA. For SkQ1 tests (*N* = 3, cell passages), there were *n* = 4, *n* = 6 (*n* = 3 for SkQ1), and *n* = 5 assays for KIC, KIV, and KMV, respectively; with *n* = 3 assays with glibenclamide. Two scrambled siRNA treatments (*N* = 2) are compared with two ETFQOR siRNA treatments (*N* = 2) and *n* = 4, *n* = 6, and *n* = 2 assays for KIC, KIV and KMV, respectively (*n* = 2 for SkQ1 at each BCKA); and *n* = 4 assays with glibenclamide. ∗∗*P* < 0.05. **B**–**E**) **Ca^2+^ oscillations with 15 mM KIC in INS-1E cells** transfected with GCaMP6s fluorescent probe – **B**,**C**,**D**) Records of GCaMP6s fluorescence from a single cell are shown. The addition of KIC is indicated by the arrow. Note that oscillations exhibited the three different patterns, i.e., with a lag phase (**B**), irregular one (**C**) and repeatable pattern (**D**). Also, inhibition by nimodipine (5 μM) is shown in panels **C**,**D**. **E**) Histogram of Ca^2+^ oscillations amplitudes sorted in their deciles of the maximum amplitude, based on 5 regions of interest (ROI) representing GCaMP6s-transfected single cells; compared to histogram of the absence of major Ca^2+^ oscillations at 3 mM glucose without BCKAs and histogram for GSIS (9 mM glucose). **F**–**K**) **Ca^2+^ oscillations with BCKAs vs. glucose in PIs isolated from heterozygous GCaMP6sβoe mice** – **F**–**I**) Records of single cells, when 10 mM KIC, KIV, and KMV or 15 mM glucose were added to isolated PIs incubated with 5.5 mM glucose (”5 mM Glc”) in an oxygenated chamber of confocal microscope. **J**,**K**) Histogram of resulting Ca^2+^ oscillations amplitudes sorted in their deciles of the maximum amplitude in PIs, based on 18 regions of interest (ROI, each equal to a single cell) for KIC, 9 ROI for KIV, 23 ROI for KMV, and 13 ROI for glucose.

Moreover, mt-targeted antioxidant SkQ1, as well as ETFQOR silencing, both inhibiting the majority of BCKA-SIS, did not allow ATP-sensitive K^+^ channels (K_ATP_) to be closed (Fig. 3A). The K_ATP_ closure was evaluated from glibenclamide-sensitive Tl^+^ fluxes, using them as a surrogate for K^+^ fluxes [14,17]. The calculated percentage of the K_ATP_ closure was more intensive for KMV > KIV > KIC at 15 mM BCKAs (Fig. 3A), thus correlating with the order of *J*^*S*^_m_ and *J*^ext^ rates (Fig. 2A–C); whereas at 1 mM KIC, it was equal to 1 mM KMV. The SkQ1-sensitive portions of Tl^+^ flux were rather similar.

All three BCKAs enabled Ca^2+^oscillations in INS-1E cells, such as evoked by KIC (Fig. 3B–E; SM Part II.3, Fig. S3A). The resulting amplitude histograms were resembling those for GSIS, but without having a peak (Fig. 3A). As expected, these Ca^2+^oscillations were blocked by 5 μM nimodipine, showing their origin from Ca_L_ channels (Fig. 3C and D). These results suggest the essential requirement of H_2_O_2_ diffusion signal for both the K_ATP_ closure and existence of Ca^2+^oscillations, upon triggering of insulin secretion.

### 3.3 Origin of H_2_O_2_ diffusion signal upon BCKA-SIS

**Fig. 4:**
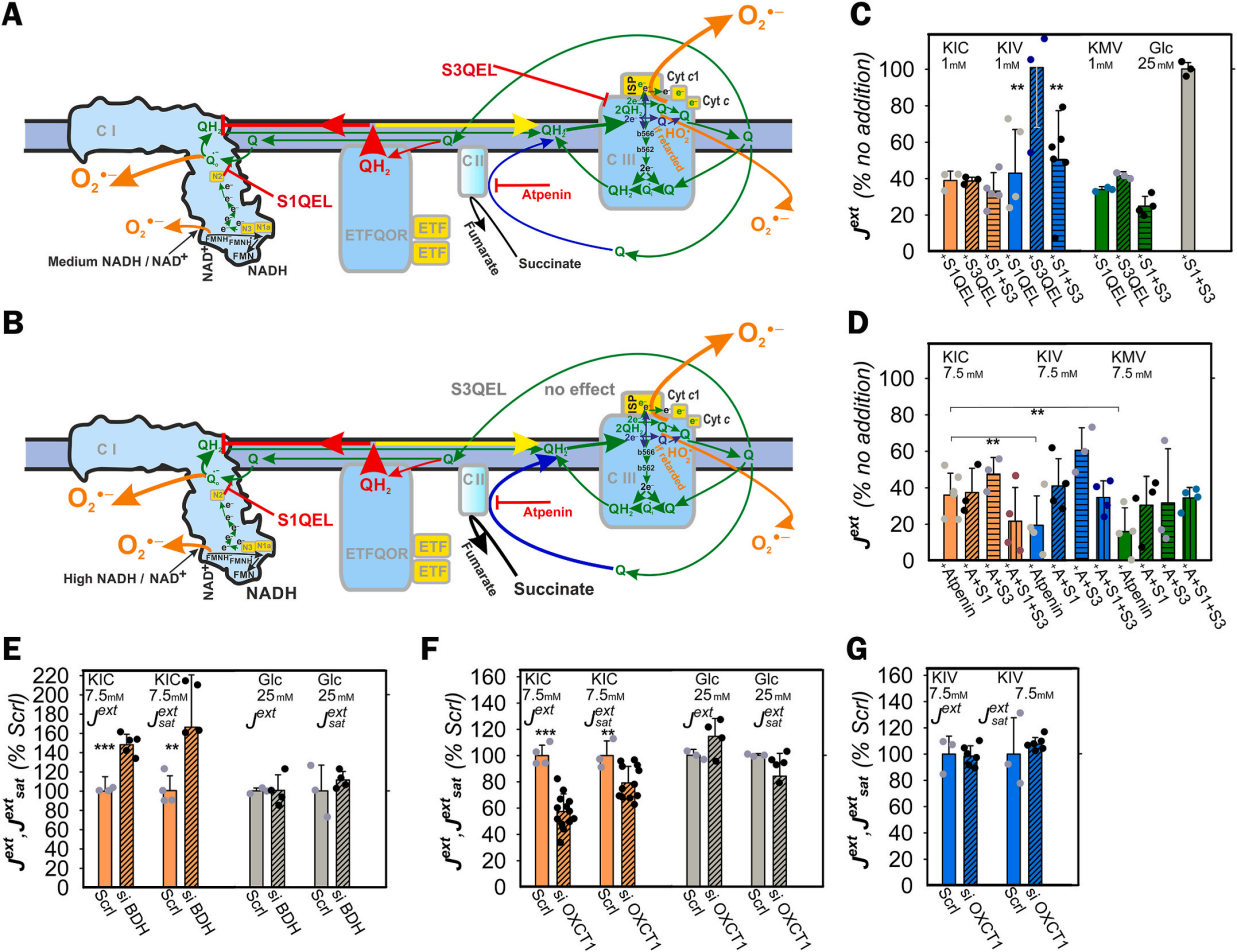
Effects on H_2_O_2_ release from INS-1E cells by site-specific antioxidants or silencing of selected enzymes – **A**–**D**) Schemes (**A**,**B**) and data (**C**) illustrating effects of S1QEL, S3QEL, and synergy of S1QEL + S3QEL for KIC- and KMV- (**A**) and for KIV- (**B**) induced extracellular H_2_O_2_ release in INS-1E cells (**C**). Note, that the scheme in panel **B** differs in assuming twice a higher substrate pressure (for NADH and succinate) relative to that considered for panel **A**. **C**) Initial rates of H_2_O_2_ release to the cell exterior (*J*_ext_) are shown after 60 min preincubation with 10 μM S1QEL, 10 μM S3QEL, and both (*N* = 3–5, *n* = 3–6). **D**) *J*_ext_ rates with 0.5 μM atpenin A5, either alone or after preincubations with 10 μM S1QEL, 10 μM S3QEL, and both (*N* = 3–4, *n* = 3–6). **E**–**G**) initial H_2_O_2_ release (*J*_ext_) rates and saturated rates (at 10 min; *J*_sat_^ext^) as induced with the indicated BCKAs and 25 mM glucose, relative to cells transfected with scrambled siRNA (*N* = 2, *n* = 3–4), in INS-1E cells silenced for 3-hydroxybutyrate dehydrogenase (**E**; *N* = 2, *n* = 4–5) and OXCT1 (**F**; *N* = 2, *n* = 5–13). For transcript and protein reduction of OXCT1 see SM, part II.5. ∗∗∗*P* < 0.001; ∗∗*P* < 0.05.

Suppressors of superoxide formation at sites I_Q_ (Complex I) and III_Q_ (Complex III), i.e., S1QEL, S3QEL, respectively [3,4,[17], [18], [19], [20], [21],^23^]), individually and both together partially inhibited the H_2_O_2_ release rates to the INS-1E cell exterior (*J*^ext^ rates) induced by BCKAs (Fig. 4A and B); however, with the exception of KIV plus S3QEL (Fig. 4C). To ensure equilibrium, we pre-incubated cells for 1 h with S1QEL, S3QEL, and both. Whereas KIC- and KMV-induced initial *J*^ext^ rates with S1QEL alone and S3QEL alone were inhibited by 60% to 65% and up to ∼70% to 75% with both together (hence any major additive effect was lacking), KIV-induced initial *J*^ext^ rates were insensitive to S3QEL. Nevertheless, the KIV-induced *J*^ext^ rates were still inhibited by 57% with S1QEL and by 55% with both. Inhibition of saturated *J*_sat_^ext^ rates with BCKAs was up to ∼70% (SM Part II.4, Fig. S4A–C; again, no inhibition existed with KIV plus S3QEL).

As an inherent control, we evaluated glucose-induced H_2_O_2_ release to the cell exterior, which exists due to the NOX4 function and hence does not originate from mitochondria [14]. As expected, S1QEL, S3QEL, and both together were ineffective towards the NOX4-induced H_2_O_2_ release (Fig. 4C).

A lower inhibitory ability relative to the ETFQOR silencing indicates that for each BCKA, S1QEL cannot completely outcompete superoxide formation. It suggests that for KIC and KMV the QH_2_ input from ETFQOR is divided into two opposite directions (Fig. 4A). A portion of superoxide release, otherwise prevented by S1QEL at site I_Q_, stems from the effective competition of the excessive ETFQOR QH_2_ input with the Complex I QH_2_ output. This competition can be viewed as the reverse electron transfer (QH_2_ transfers electrons from ETFQOR to Complex I), producing superoxide at site I_Q_ [3]. S1QEL withdraws such competition by redirecting excessive electrons to itself and hence superoxide cannot be formed at site I_Q_ [18,19]. Simultaneously, the other portion of the ETFQOR QH_2_ input merges with the forward electron transfer towards Complex III. Resulting excessive electron input causes the S3QEL-sensitive superoxide formation at the Complex III site III_Qo_. Finally, with both S1QEL plus S3QEL, the remaining 20–30% superoxide formation could originate from the flavin site I_F_ of Complex I and other sites.

For KIV β-like oxidation, the excessive succinyl-CoA and following succinate input to Complex II/SDH plus a portion of ETFQOR electron (QH_2_) input provide reverse electron transfer to Complex I site I_Q_ and another congestion of QH_2_ fluxes before entering the Q-cycle of Complex III. As a result, superoxide is formed at sites I_Q_ and III_Qo_, respectively. Apparently, the downstream S3QEL action cannot withdraw such congestion (electron transfer retardation), so it does not inhibit superoxide formation at site III_Qo_ and concomitant H_2_O_2_ release for KIV.

Moreover, since the KIV β-like oxidation yields three NADH molecules, a maximum among BCKAs (Fig.1; Fig. 4B), the resulting high NADH_mt_/NAD ^+^_ mt_ ratio causes much higher superoxide formation at site I_F_ relative to KIC. In addition, the downstream forward electron (QH_2_) flux via site I_Q_ encounters the ETFQOR-excessive QH_2_ and provides the superoxide formation, which is otherwise withdrawn by S1QEL. It is possible that the sum of excessive electron (QH_2_) fluxes from the elevated NADH, ETF and succinate inputs before the site III_Qo_ is so high that the S3QEL cannot compete and is nearly ineffective. Finally, KMV has two inputs to the Krebs cycle, acetyl-CoA as well as succinyl-CoA and behaves as a “mix” of KIC and KIV.

To investigate further the Complex II role, we used atpenin A5 as the strongest Complex II/SDH inhibitor, known not to induce superoxide release at the SDHD subunit [22]. Thus, 0.5 μM atpenin A5 was also partially inhibiting the initial *J*^ext^ rates for KIC (by 65%) and strongly inhibited them for KIV (by 80%) and KMV (by 85%) (Fig. 4D; for *J*_sat_^ext^ rates see Fig. S4D). Atpenin A5 by interrupting Complex II QH_2_ input prevents the QH_2_ congestion in front of Complex III and hence does not allow superoxide formation and following H_2_O_2_ release (Fig. 4A and B). This effect is stronger with KIV and KMV, due to the withdrawal of much higher succinate pressure and possible feedback retardation of the Krebs cycle, releasing the NADH substrate pressure and superoxide formation at sites I_F_ and I_Q_.

For KIC and KMV, preincubations with S1QEL alone, S3QEL alone, (for KMV also with both S1QEL + S3QEL), followed by atpenin A5 addition, did not further attenuate the initial *J*^ext^ rates (Fig. 4D; Fig. S4D). The lack of synergy with S1QEL + S3QEL demonstrated that the possible feedback retardation of Krebs cycle with atpenin A5 and the resulting block of the QH_2_ input by succinate cannot further withdraw any excessive electron transfer retardation. Nevertheless, in intact cells Complex II/SDH contributes to the overall superoxide formation by congestion of QH_2_ fluxes before entering Complex III. Also, after pre-incubation with S1QEL, S3QEL, or both atpenin A5 lost its inhibitory ability for KIV (apparently for KMV), i.e., lost its antioxidant action. This reflects that the withdrawal of QH_2_ input congestion in front of Complex III by atpenin A5 is not sufficient to prevent superoxide formation due to the QH_2_ input by ETFQOR (otherwise bifurcated to the two directions).

### 3.4 Effects of auxiliary reactions upon BCKA β-like oxidation and BCKA-SIS

For H_2_O_2_ release induced by 7.5 mM KIC, silencing of 3-hydroxybutyrate dehydrogenase (BDH) did increase *J*^ext^ and *J*_sat_^ext^ rates (Fig. 4E; SM Part II.5, Fig. S4E), indicating a higher NADH yield at BDH deficiency and resulting higher superoxide formation at the flavin site I_F_. This is because the uninhibited BDH reaction requires NADH for conversion of acetoacetate to 3-hydroxybutyrate, further diminishing the NADH yield (Fig. 1). Note, BDH silencing did not affect *J*_m_ and *J*_sat_^ext^ rates induced by 25 mM glucose (Fig. 4E), since NOX4 is responsible for such a H_2_O_2_ signaling [14].

In contrast, silencing of 3-oxoacid-CoA-transferase-1 (OXCT1/SCOT1, aka succinyl-CoA-3-oxaloacid-CoA transferase) (Fig. S4F–H) decreased *J*_m_ and *J*_sat_^ext^ rates with 7.5 mM KIC by 40% and 20%, respectively (Fig. 4F). This shows that the excessive succinate produced by OXCT1/SCOT1 partly contributes to the reverse electron transfer and induces more superoxide/H_2_O_2_ in controls transfected with scrambled siRNAs. Here again, marginal insignificant changes in *J*_m_ and *J*_sat_^ext^ rates occurred with 25 mM glucose as well as with 7.5 mM KIV, where the OXCT1/SCOT1 reaction is absent (Fig. 4F and G).

### 3.5 Unbiased metabolomics supports the existence of BCKA β-like oxidation upon BCKA-SIS in INS-1E cells

**Fig. 5:**
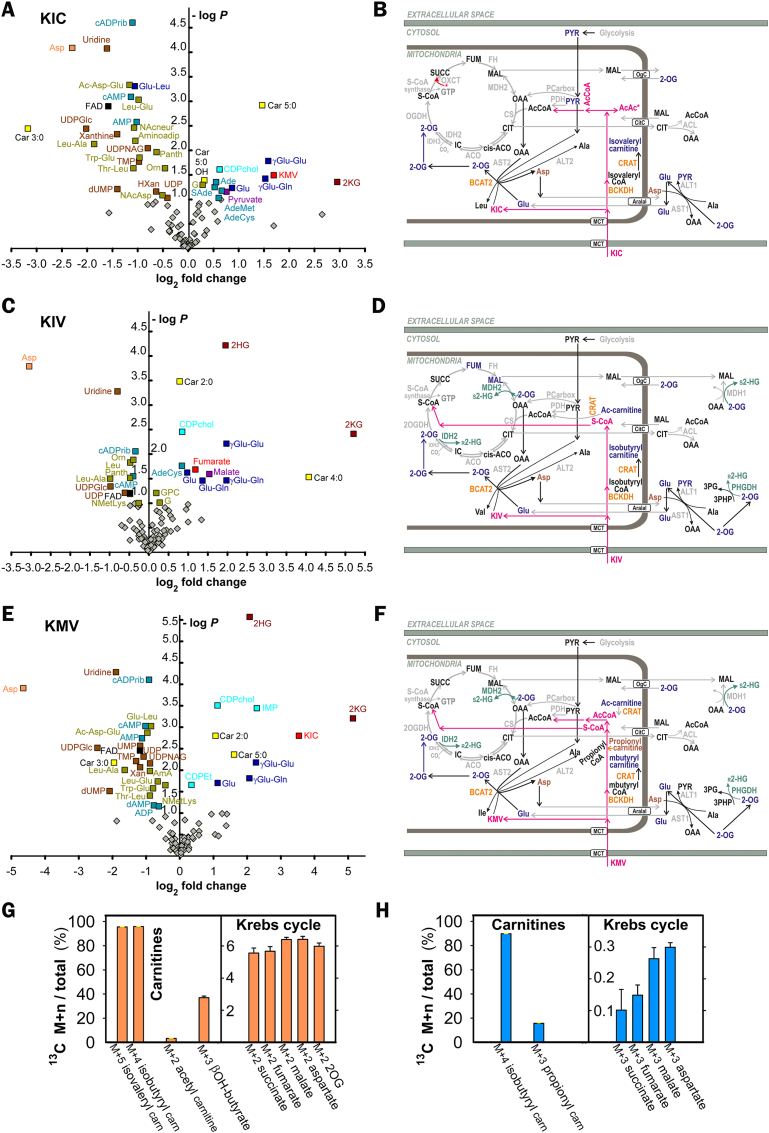
**Metabolomics analyses of β-like oxidation of BCKAs in INS-1E cells** – **A**–**C**) Polar metabolites (*N* = 6) revealed 1 h after incubation with 15 mM BCKAs; **D**–**F**) interpretation of results, assuming that rat INS-1E cells do not express BCAT1, since its presence was found only in the brain, ovaries and testes [1]. **G**,**H**) **KIC and KIV metabolism surveyed with U-^13^C-KIC and U-^13^C-KIV**, respectively – tracking of M+5, M+4, M+3 and M+2 isotope species, expressed in % of the total isotopes of the particular compound, after 1 h incubation of INS-1E cells in KRH buffer with 7.5 mM U-^13^C-KIC and 7.5 mM U-^13^C-KIV in the presence of 1 mM l-carnitine, 3 mM glucose, 2 mM glutamine, and 0.1% BSA (*N* = 2).

Unbiased polar metabolomics analyses for oxidation of individual 15 mM BCKAs, ongoing for 1 h in INS-1E cells (Fig. 5A–C,E), confirmed theoretical predictions for metabolic modes (Fig. 5B–D,F), where KIC, KIV, or KMV β-like oxidation dominates over glycolysis and glutaminolysis. Thus, β-like oxidation of each individual BCKA led to the decrease (marked with *brown* fonts) of aspartate and provided the increase or accumulation (*dark blue* fonts) of 2-ketoglutarate (2-oxoglutarate, 2-OG) and glutamate (Fig. 5A–C,E). This confirms that the presumed aminotransferase reactions in the matrix (ALT2, AST2, and BCAT2) run in the direction of 2-OG synthesis, utilizing excessive glutamate. In contrast, cytosolic ALT1 and AST1 synthesize glutamate and AST1 cause the observed aspartate decline. If the BCAT2 reaction was in effect, it would decrease BCKA β-like oxidation.

The existing KIC β-like oxidation was suggested by the increase in total C5-carnitines (Fig. 5A; unresolved for BC-carnitines), that might include isovaleryl carnitine, coming from the carnitine-O-acetyltransferase- (CRAT-) mediated conversion of isovaleryl-CoA. The latter is the ultimate reaction product of BCKDH. Pyruvate was increased upon KIC oxidation, indicating the active ALT1 reaction and possible competition with the additional acetyl-CoA input into the Krebs cycle. Also, dipeptides γGlu-Glu or γGlu-Gln and CDP-choline were accumulated.

Analogously, the β-like oxidation of KIV was evidenced by the increase of total C4-carnitines and C2-carnitine (acetyl-carnitine; Fig. 5C). Among C4-carnitines, isobutyryl-carnitine might be included as a reaction product of CRAT, converted from isobutyryl-CoA. The latter is again the BCKDH reaction product. Moreover, a significant 2-hydroxyglutarate (2-HG [28]) accumulation suggested possible 2-HG creation be several enzymes under these conditions (SM Part II.6). Existing KMV oxidation exhibited features of both previous metabolic schemes (Fig. 5E). The increase of total C5-carnitines could include 2-methylbutyryl carnitine („mbutyryl“) produced by CRAT from the BCKDH-product 2-methylbutyryl-CoA. The observed 2-HG accumulation can be interpreted identically as for KIV.

Our metabolomics analyses also revealed a cross-direction into the KMV pathway upon KIC β-like oxidation, as well as an overflow to the KIC pathway upon KMV β-like oxidation. This indicates that a slightly accumulated pyruvate represents a cross-point, promoting synthesis of isoleucine (KIC to KMV direction) and leucine (KMV to KIC direction). KMV and KIC, respectively, serve as the last-but-one intermediates in these syntheses. Finally, unbiased lipidomic analyses made after 1-h oxidation of individual BCKAs did not show major changes relative to the initial time of reaction (SM Part II.6, Fig. S5A–C).

### 3.6 Tracing of ^13^C-metabolites downstream of BCKA β-like oxidation determining BCKA-SIS

To confirm definitively the existence of BCKA β-like oxidation in INS-1E cells and survey auxiliary reactions, we employed U-^13^C-KIC (Fig. 5G) and U-^13^C-KIV (Fig. 5H) for identification of ^13^C-labeled downstream products. Carnitines were surveyed as reaction products of CRAT, which converts them from the respective R–CoAs [26]. After 1-h incubation of INS-1E cells with 7.5 mM U-^13^C-KIC, the M+5 intermediates were found, such as M+5 isovalerylcarnitine accounting for 95.5% ± 0.1% of total isovalerylcarnitine isotopes (complying with natural ^12^C-KIC, which accounted initially for ∼4%) and M+5 methylcrotonylcarnitine (96% ± 0.1% of total), as well as M+3 3-hydroxybutyrate (37% ± 1% of total isotopes). In addition, M+2 acetyl-carnitine attained 3.3% ± 0.1% of total isotopes and M+1 acetyl-carnitine was 3.9% ± 0.4% of total isotopes, together being splitting products of acetoacetylthiolase (Fig. S1A).

M+2 Krebs cycle intermediates amounted up to 6% of total individual intermediates, including M+1 isocitrate being 3.3% ± 0.2% of total isocitrate. Enriched M+2 aspartate could arise from the AST2 reaction, consuming oxaloacetate (Fig. 5B). These results perfectly reflect theoretical predictions for the complete β-like oxidation of U-^13^C-KIC (Fig. S1A), when carnitines, being reaction products of CRAT [26]. Also, the OXCT1 reaction and BDH production of 3-hydroxybutyrate were confirmed.

After 1-h incubation of INS-1E cells with 7.5 mM U-^13^C-KIV, M+4 isobutyrylcarnitine accounted for 90.0% ± 0.1% of total isobutyrylcarnitine isotopes and M+3 propionylcarnitine for 15.7% ± 0.5% of total propionylcarnitine (Fig. 5H). Note that again the identified carnitines were reaction products of CRAT [26]. Moreover, M+3 malate, M+3 fumarate, and M+3 succinate were detected after U-^13^C-KIV oxidation, reflecting theoretical predictions (Fig. S1B). Enriched M+3 aspartate could arise from the AST2 reaction, consuming oxaloacetate (Fig. 5D). The absence of M+4 metabolites of the Krebs cycle indicated that the branch including ALDH2-ACSF3-MMUT reactions has a negligible contribution (Fig. 1; Fig. S1B).

### 3.7 Branched-chain keto acid-stimulated insulin secretion in pancreatic islets

**Fig. 6:**
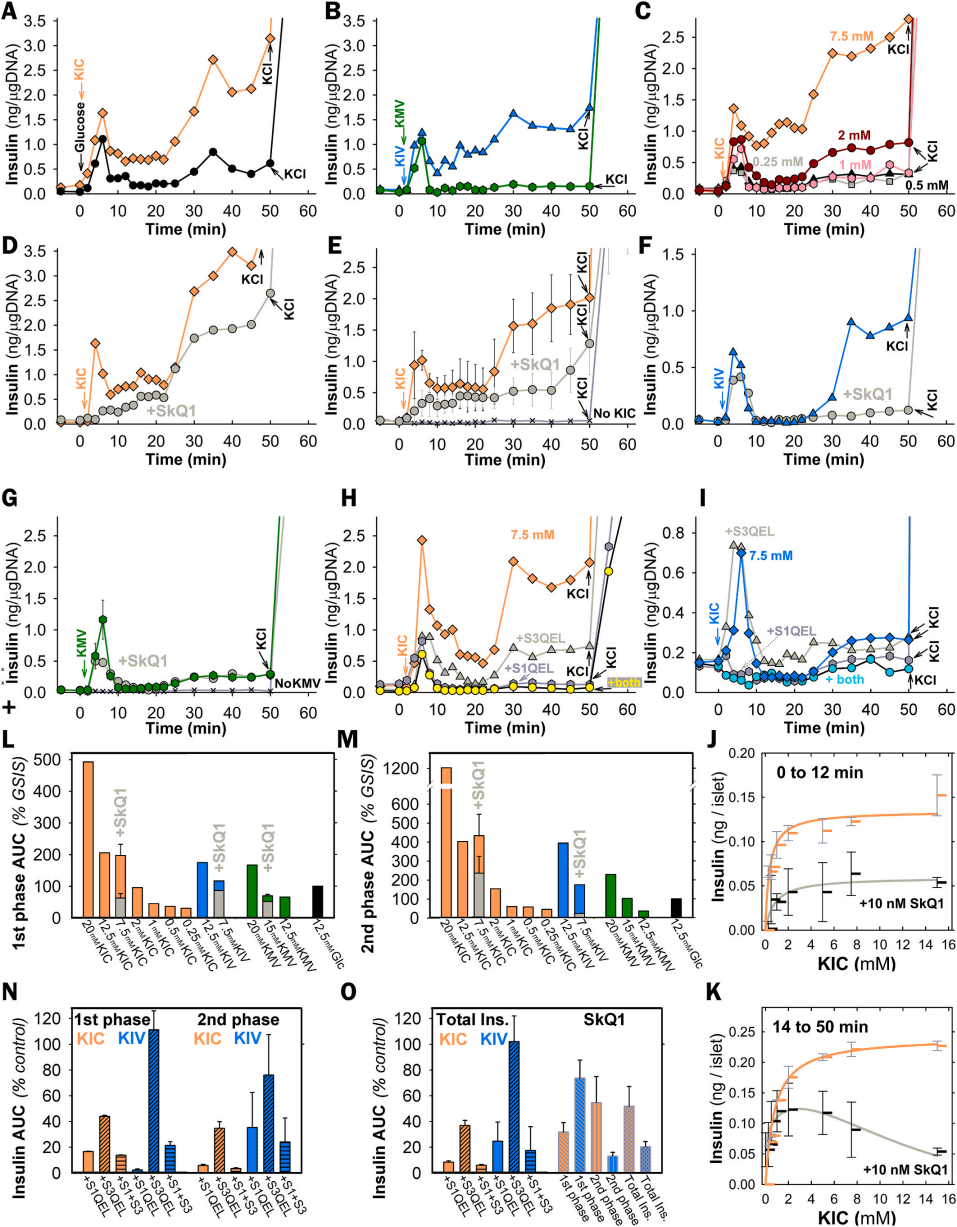
**Insulin secretion in perifused pancreatic islets stimulated with BCKAs** – **A**–**I**) Time course of insulin release rates (amounts released in previous 2 or 5 min) from perifused PIs with 5.5 mM glucose exposed to 12.5 mM KIC (**A**; *orange*); a series of KIC doses from 0.25 mM to 7.5 mM (**C**); 7.5 mM KIC (**D**,**E,H**); 12.5 mM KIV (**B**; *blue*); 7.5 mM KIV (**F,I**) and 12.5 mM KMV (**B**,**G**; *green*). Each panel represents a distinct PI isolation (when SD bars present *N* = 2). Panel **A** (*black*) also includes 12.5 mM glucose alone for comparison. Where indicated, PIs were preincubated for 10 min with 100 nM SkQ1 (**D**–**G**) and then perifused in the continued presence of 10 nM SkQ1, which was also used for static incubations (**J**,**K**). **H**,**I**) Rates of insulin release with time during PI perifusions with 7.5 mM KIC (**H**; *orange*) and 7.5 mM KIV (**I**; *blue*) in the presence of 2 μM S1QEL, 2 μM S3QEL or both (2 μM each) as indicated. **J**,**K**) Total insulin accumulation during static incubations of PIs at the indicated time points. **L**,**M**) Total insulin release estimated from areas under the curve (AUCs, *N* = 30; for 7.5 mM KIC, *N* = 9) for the 1st phase (**L**) and the 2nd phase of secretion (**M**). Inhibition after preincubations with 100 nM SkQ1 is shown as the *gray* portion of the bars (*N* = 3). **N**,**O**) Inhibition by S1QEL, S3QEL, or both antioxidants expressed as the remaining percentage of AUCs, separately for the 1st and 2nd phases (**N**; *N* = 3) and for total insulin AUC (combined phases; **O**). The *right-hand* part of panel **O** additionally shows inhibition by SkQ1.

**Fig. 7:**
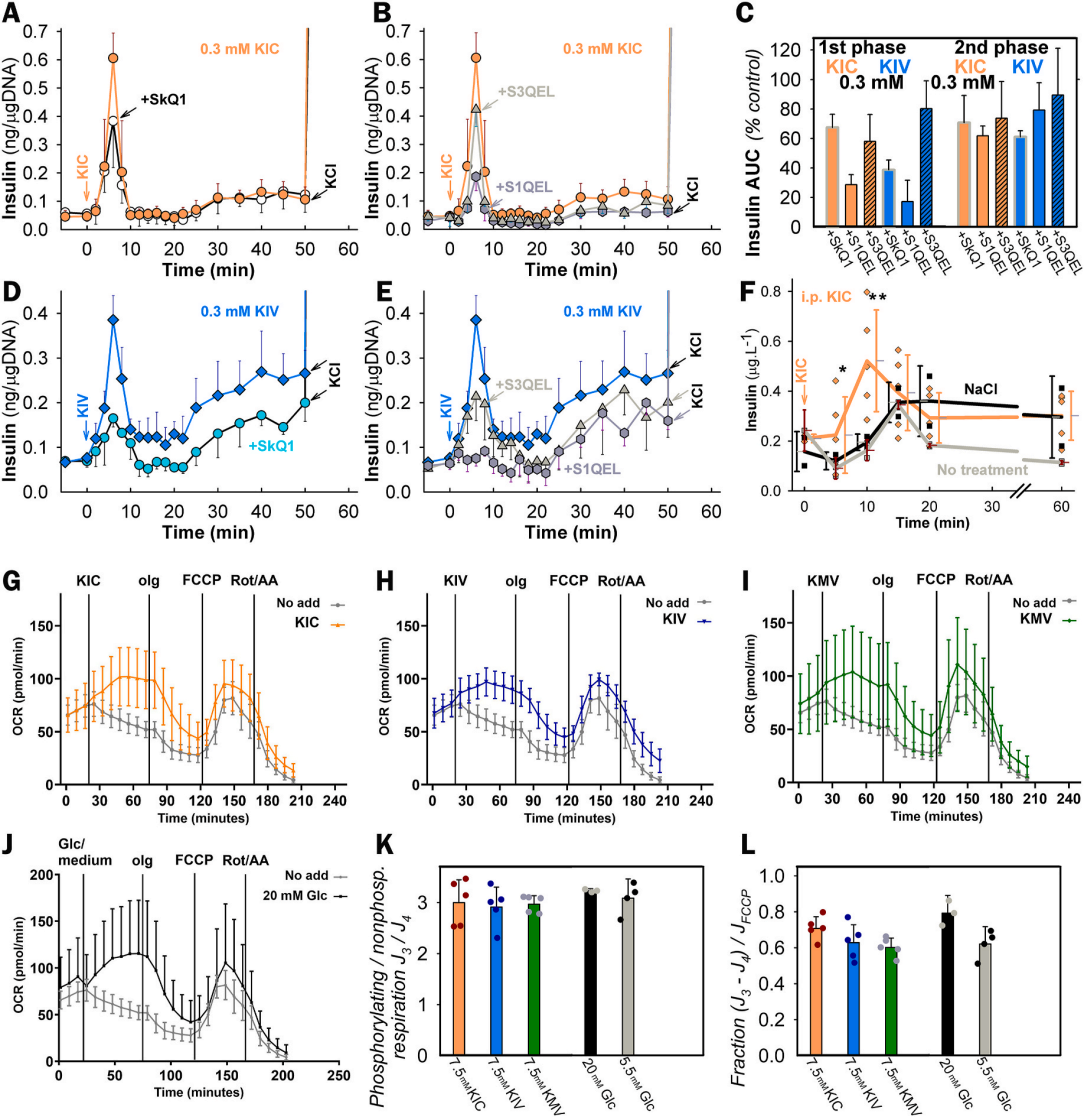
**BCKA-SIS at 0.3 mM KIC and KIV in PIs, its simulation in mice, and pancreatic islet respiration with BCKAs** – **A**–**E**) Time course of insulin release rates (amounts released in previous 2 or 5 min) from perifused PIs with 5.5 mM glucose exposed to 0.3 mM KIC (**A**,**B**; *orange*) and 0.3 mM KIV (**D**,**E**; *blue*); with SDs of *N* = 3 distinct PI isolations. Panels **A**,**D** (*black*) also include PI preincubations for 10 min with 10 nM SkQ1 and perifusion with the same dose while panels **B**,**E** preincubations/perifusion with presence of 2 μM S1QEL and 2 μM S3QEL. **C**) Resulting relative AUCs. **D**) Insulin in murine blood after i. p. administration of 10 mg KIC per kg, compared to i. p. administration of 0.9% NaCl to mice or after no treatment (for glycemia time-course see Fig. S8). ∗∗*P* < 0.05, ∗*P* < 0.1. **G**–**H**) PI respiration as revealed by typical Agilent Seahorse XF analyzer runs (averages, SDs from *n* = 6 measurements; number of PI isolations *N* = 3) are shown for **A**) 7.5 mM KIC; **B**) 7.5 mM KIV; **C**) 7.5 mM KMV; **D**) 20 mM glucose; and **E**) just a medium with resting glucose (5.5 mM, i.e., inducing no insulin secretion), adjusted to assay respiration of approximately 50 islets. **F**) Parameters derived from *n* = 6 measurements (*N* = 3) for each species are summarized, i.e., *J*_3_/*J*_4_ phosphorylating (*J*_3_) to non-phosphorylating respiration (*J*_4_) ratio and fractions (*J*_3_ - *J*_4_)/*J*_FCCP_ (in %), i.e., portions from the maximum respiration capacity, which are devoted to OXPHOS.

Next, we estimated insulin secretion in isolated murine pancreatic islets (PIs) at 5.5 mM glucose, alone not inducing insulin secretion. Continuously, instant insulin secretion rates evaluated during PI perifusion at each time point of BCKA-SIS with 12.5 mM BCKAs were maximum for KIC in both phases (2nd-phase started spontaneously at ∼20 min) (Fig. 6A), reached intermediate values for KIV (Fig. 6B), and the slowest ones for KMV (Fig. 6B). Similar order of insulin secretion rates was found for 7.5 mM (Fig. 6C–I) and 0.3 mM BCKAs (Fig. 7A–E; Fig. S7A and B). Dose-responses for insulin assays with static PI incubations (Fig. 6J and K) showed that 0.3 mM concentrations, existing in vivo, account for ∼50% of maximum rates (also cf. Fig. 6A vs. 7A; Fig. 6B vs. 7D).

To estimate the total secreted insulin, we calculated areas under curves (AUCs) of perifusion records. AUCs were decreasing in the order of KIC > KIV > KMV (Fig. 6L and M). Also, KIC dose-response is illustrated in Fig. 6C, starting with 0.25 mM dose, when it yields about one third of AUCs for 12.5 mM glucose (GSIS). KMV stimulated insulin secretion with a minimum 2nd-phase at 12.5 mM (Fig. 6B). Nevertheless, the 2nd-phase was apparent at racemic 15–20 mM KMV (i.e., 7.5–10 mM l-KMV; Fig. 6G–M). For KIC and KIV, the insulin release rates were still faster and AUCs greater than for PI perifusion with 12.5 mM glucose (GSIS), while for KMV they were similar during the 1st-phase.

Preincubations for 10 min with 10 nM (Fig. 6J and K; Fig. 7A–C,D) and 100 nM SkQ1 (Fig. 6D–G) inhibited insulin secretion stimulated with 7.5 mM (Fig. 6D,E,L,M,O) and 0.3 mM KIC (Fig. 7A–C) in perifused PIs and similarly with KIV (Fig. 6F–L,M,O; Fig. 7C and D) and KMV (Fig. 6G–L,M; Fig. S7A and B). Insulin secretion rates and AUCs in PIs perifused with 2 μM S3QEL were partially inhibited when stimulated with 7.5 mM (Fig. 6H–N,O) and 0.3 mM KIC (Fig. 7C), but not with 7.5 mM (Fig. 6I–N,O) and 0.3 mM KIV (Fig. 7C). This result complies with the inability of S3QEL to inhibit KIV-induced H_2_O_2_ release from cells (Fig. 4C). When PIs were stimulated with BCKAs and were perifused also with 2 μM S1QEL or S1QEL + S3QEL (both 2 μM), insulin secretion rates at peaks of the 1st phase were partially inhibited, but the entire or phase-related AUCs were nearly completely inhibited (Fig. 6H,I,N,O). In conclusion, mitochondria-targeted antioxidants inhibited secretion in perifused PIs at 7.5 mM, as well as 0.3 mM BCKAs (Fig. 7A–E; Fig. S7A and B), indicating the identical mechanisms.

### 3.8 Branched-chain keto-acid-stimulated insulin secretion depends on Ca^2+^oscillations in pancreatic islets

Ca^2+^oscillations at low 5.5 mM glucose were evoked by BCKAs in PIs of GCaMP6sβoe heterozygous mice (Fig. 3F–K). Ca^2+^oscillations induced in PIs by 10 mM KIC were sustained (Fig. 3F), similarly to those evoked by 15 mM glucose (Fig. 3I). This was reflected by the nearly identical intensity histograms with Ca^2+^oscillation amplitudes spread over the entire intensity scale, peaking at intermediate amplitudes (Fig. 3J). The resting state, when no insulin secretion took place, such as at 5.5 mM glucose in PIs (3 mM with INS-1E cells), was reflected by the lowest decile Ca^2+^ oscillation amplitudes (Fig. 3E–J; *gray*). Indeed, GSIS exhibited similar patterns as BCKA-SIS (Fig. 3E–J; *black, thin bars*).

In contrast, Ca^2+^oscillations induced by 10 mM KIV (Fig. 3G–K) and 10 mM KMV (Fig. 3H–K) exhibited lag phases, which are typically observed with intermediate glucose [12]. Lag phases were reflected in the corresponding histograms as frequent occurrence of the lowest decile amplitudes (Fig. 3K).

### 3.9 Branched-chain keto acid-stimulated insulin secretion depends on mt-plasma membrane H_2_O_2_ signal in PIs

**Fig. 8:**
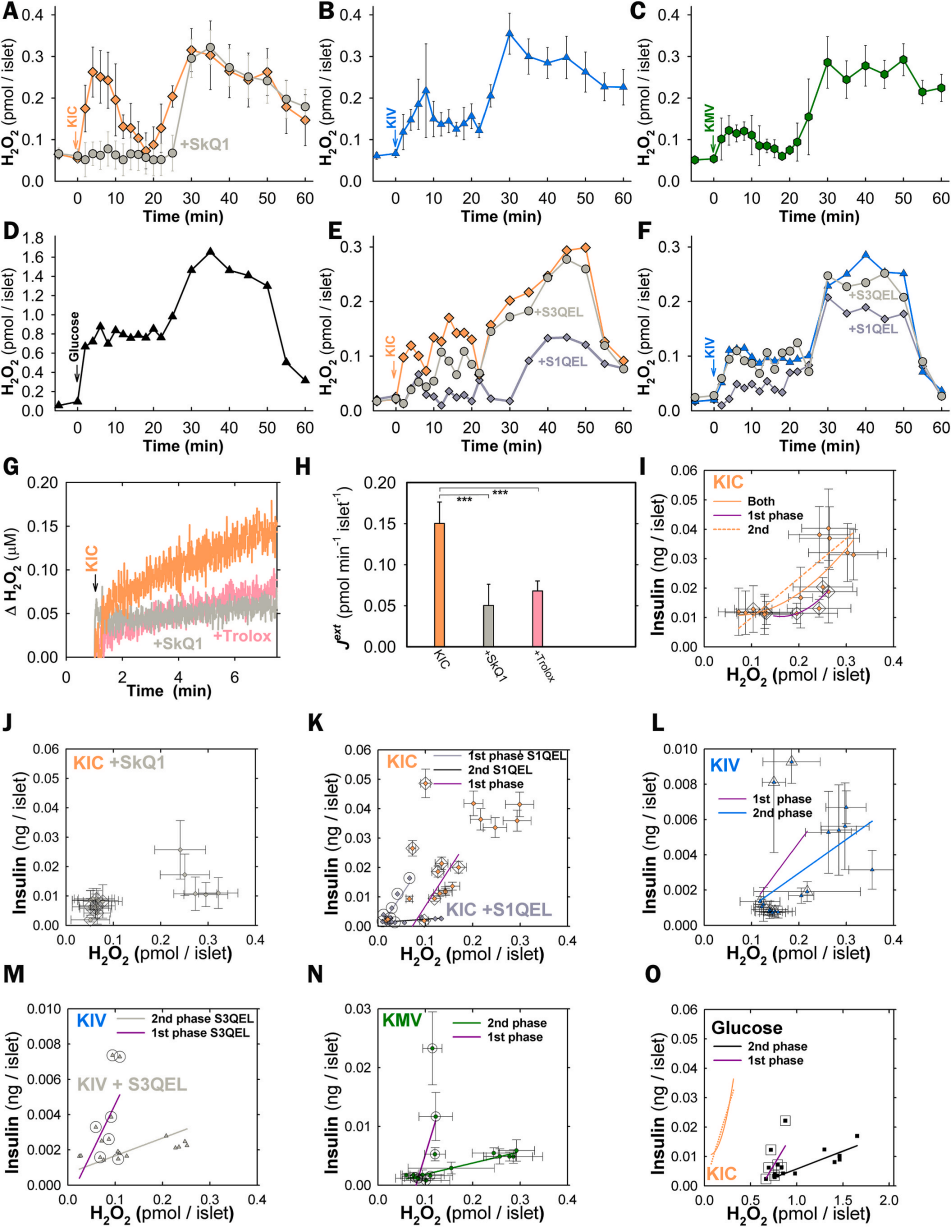
**H_2_O_2_ release from perifused pancreatic islets induced by BCKAs** – **A**–**F**) Time course of rates of H_2_O_2_ release to the extracellular medium (amounts released in previous 2 or 5 min; *N* = 3) during perifusion of PIs with 7.5 mM KIC (**A**,**E**; *orange*), 7.5 mM KIV (**B**,**F**; *blue*), 15 mM racemic KMV (**C**; *green*) and 7.5 mM glucose (**D**; *black*). Where indicated, PIs were preincubated for 10 min with 100 nM SkQ1, and then perifused in the continuous presence of 10 nM SkQ1. Where indicated, 2 μM S1QEL and 2 μM S3QEL were included in the perifusion medium (**E**,**F**). **G**,**H**) Static assay of H_2_O_2_ release induced by 7.5 mM KIC in the presence of Amplex UltraRed and HRP. Representative traces (**G**) and corresponding quantifications (**H**; *N* = 2) are shown, including conditions with 10 nM SkQ1 and 100 μM Trolox; ∗∗∗*P* < 0.001. **I**–**O**) Correlation between insulin secretion rates (from Fig. 6, panels **D**,**F**,**G**) and the corresponding rates of H_2_O_2_ release to the extracellular medium. Data were fitted separately for the 1st phase of insulin secretion (2 to 12 min; data with large *black contours*) and the 2nd phase (14 to 50 min; data *without contours*) as indicated by legends.

**Fig. 9:**
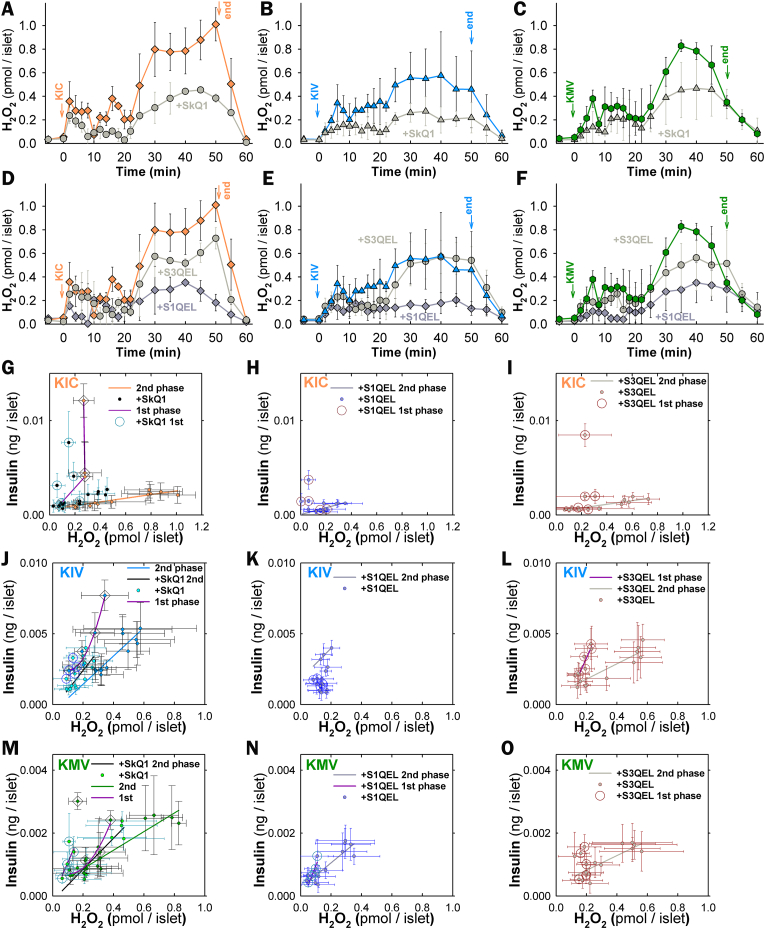
**H_2_O_2_ release from perifused pancreatic islets induced by 0.3 mM BCKAs** – **A**–**F**) Time course of rates of H_2_O_2_ release to the extracellular medium (amounts released in previous 2 or 5 min; *N* = 4) during perifusion of PIs with 0.3 mM KIC (**A**,**D**; *orange*), 0.3 mM KIV (**B**,**E**; *blue*), and 0.3 mM racemic KMV (**C**,**F**; *green*). Arrows with “end” indicate the moment when BCKAs were withdrawn from the perifusate. Where indicated, PIs were preincubated for 10 min with 100 nM SkQ1, and then perifused in the continuous presence of 10 nM SkQ1 (**A**–**C**). Where indicated, 2 μM S1QEL and 2 μM S3QEL were included in the perifusion medium (**D**–**F**). **G**–**O**) Correlation between insulin secretion rates (data from Fig. 7, panels **A**,**B**,**D**,**F** and Fig. S7, panels **A**,**B**) and the corresponding rates of H_2_O_2_ release to the extracellular medium (data from panels **A**–**F**). Data were fitted separately for the 1st phase of insulin secretion (2 to 10 min; data with large diamond and circle *contours*; fits in *violet*) and separately the 2nd phase (12 to 50 min; data *without contours*; fits as indicated in legends).

Next, we examined the relationship between the H_2_O_2_ diffusion signal and insulin secretion in isolated pancreatic islets (PIs) at 5.5 mM glucose, which alone did not induce insulin secretion. The existence of a mt-PM H_2_O_2_ signal during BCKA-SIS was demonstrated using Amplex UltraRed plus HRP in PI perifusates, where the probe did not directly contact the islets. In this way, H_2_O_2_ released to the extracellular compartment was monitored (Fig. 8A–C; E,F; Fig. 9A–F; for NOX4-originating H_2_O_2_ with glucose see Fig. 8D). The maximum observed BCKA-stimulated H_2_O_2_-release rates to the PI exterior for the 1st phase at 5.5 mM glucose and 7.5 mM BCKAs were 87 ± 20, 73 ± 38, and 41 ± 12 pmol min^−1^·10^−6^ PI-cells for KIC, KIV, and KMV, respectively; considering 1500 cells per single islet. These values can be compared with 290 ± 30 for 7.5 mM glucose, where NOX4 produces H_2_O_2_ (Fig. 8D); or to 0.3 mM KIC (92 ± 40 pmol min^−1^·10^−6^ PI-cells; Fig. 9A). A biphasic pattern paralleling biphasic insulin exocytosis suggested that H_2_O_2_ reached the cell exterior in temporal association with insulin granule exocytosis. Maximum H_2_O_2_-release rates for BCKAs in the 2nd phases ranged between 100 and 300 pmol min^−1^·10^−6^ PI-cells (550 for GSIS; Fig. 8D).

The extracellular H_2_O_2_ release upon BCKA-SIS in PIs was also predominantly blocked by SkQ1, when tested in dynamic perifusions (Fig. 8A–J; Fig. 9A–C; G,J,M) or static incubations (Fig. 8G and H). Trolox, a water-soluble vitamin E analog, exerted the same effect (Fig. 8G and H). Strong, but incomplete, inhibition of the extracellular H_2_O_2_ release during the 1st phase and partial inhibition during the 2nd phase was observed with 2 μM S1QEL (Fig. 8E,F,K; Fig. 9D–F; H,K,N). A partial inhibition by 2 μM S3QEL was recognized only for KIC and KMV, but not KIV (Fig. 8E and F; Fig. 9D–F; I,L,O). This result again confirms in PIs the obtained results in INS-1E cells, i.e., the inability of S3QEL to inhibit KIV-induced H_2_O_2_ release (Fig. 4C). Collectively these results demonstrate that the mt-PM H_2_O_2_ diffusion signal is linked to BCKA-SIS.

Such essential dependence was further evidenced by non-constant relationships between insulin secretion rates and extracellular H_2_O_2_ release rates at each time point (Fig. 8I–O; Fig. 9G–O). The two phases were clearly distinguished. A steep relationship between insulin secretion rates and extracellular H_2_O_2_ release rates was found for the triggering 1st phase. Less steep correlation was obtained for the 2nd phase (14 to 50 min) of BCKA-SIS as well as for glucose (GSIS) (Fig. 8I–O; Fig. 9G–O; SM Part II.7, Fig. S6A–F). Separate fits of each phase showed not only the evident correlation but also left-shifts of these dependencies with antioxidants, i.e., by SkQ1, S1QEL, and S3QEL. The non-linearity and steepness of these 1st phase relationships suggest that with any further incremental H_2_O_2_ surplus/accumulation a higher portion of insulin granule vesicles is promoted for exocytosis. We remain here that in the 1st phase only vesicles already positioned near the plasma membrane undergo exocytosis.

Note that the mitochondrial H_2_O_2_ release proceeded at one order of magnitude lower levels relative to the NOX4 H_2_O_2_ release, stimulating GSIS (Fig. 8O). More importantly, the 1st phase insulin/H_2_O_2_ stoichiometry for GSIS and KIC-stimulated-insulin secretion were within the same order of magnitude, indicating a shared redox-triggering principle (Fig. 8I–O; Fig. 9G–O; Fig. S6A–F), despite the different H_2_O_2_ origin (NOX4 vs. mitochondrial). The apparent maximum stoichiometry at the 1st phase was ∼0.15 and > 0.05 ng insulin per pmol H_2_O_2_ for 7.5 mM and 0.3 mM KIC, respectively. Compare it to 0.054 ng insulin per pmol H_2_O_2_ for 7.5 mM glucose. These stoichiometries for 7.5 mM KIV and KMV attained values of 0.44 and 0.27 ng insulin per pmol H_2_O_2_ or 0.04 and 0.01 at 0.3 mM.

For the 2nd phase, the stoichiometry was ∼0.1 and 0.003 ng insulin per pmol H_2_O_2_ for 7.5 mM and 0.3 mM KIC, respectively; compared to 0.012 for glucose. For 7.5 mM KIV and KMV, 2nd phase stoichiometry values reached 0.1 and 0.02 at 7.5 mM or 0.01 and 0.003 at 0.3 mM. The lower stoichiometries at 0.3 mM BCKAs may reflect the lowered yield of ATP. Undoubtedly, ATP (ATP/ADP) elevation is also required together with H_2_O_2_ for a closure of K_ATP_ channels.

### 3.10 Branched-chain keto acids increase OXPHOS in pancreatic islets

PI respiration with 7.5 mM BCKAs at 5.5 mM glucose exhibited mitochondrial coupling parameters reflecting ongoing OXPHOS (Fig. 7G–L). The average phosphorylating (*J*_3_) to non-phosphorylating respiration (*J*_4_) ratio (*J*_3_/*J*_4_) was around 3, slightly lower than with 20 mM glucose (Fig. 7K). Note, the non-phosphorylating respiration was estimated with 5 μM oligomycin. Also, a fraction relative to the maximum respiration capacity, which is devoted to OXPHOS, i.e. (*J*_3_ - *J*_4_)/*J*_FCCP_ (in %), was 71% for KIC, 63% for KIV, and 60% for KMV, as compared to 80% and 60% for 25 mM and 5 mM glucose, respectively (Fig. 7L).

### 3.11 In vivo simulation of KIC-stimulated insulin secretion in mice

Our simulations of BCKA-SIS in mice used intraperitoneal (i.p.) injections of 10 mg/kg KIC and measurements of time-course of blood insulin and glucose in a cohort of mice, similarly, as performed for intralipid [17] and glucose [14]. Three time points were assayed for each individual mouse and resulting data, combined for the entire cohort, were compared with those of iso-osmolar NaCl injections and no treatment (Fig. 7L; Fig. S7A). Similarly, as for glucose (GSIS), after KIC administration insulin peaked at ∼10 min, declining up to 60 min.

## 4 Discussion

In this study, using murine PIs and rat INS-1E β-cells, we demonstrated that oxidation of each of three BCKAs increases not only ATP production, but also mitochondrial superoxide/H_2_O_2_. The resulting mitochondria-to-plasma-membrane H_2_O_2_ diffusion signal promotes K_ATP_ channel closure and thereby stimulates insulin secretion. The K_ATP_ channels are closed most probably due to an intrinsic redox change. The source for such H_2_O_2_ signaling with BCKAs was identified to be of mitochondrial origin. It was successfully monitored as the H_2_O_2_ release to the cell/PI exterior [17]. The mitochondrial origin was evidenced by prevention of the majority of H_2_O_2_ release in cells deficient of mitochondrial protein ETFQOR, as well as upon inhibition by mitochondria-targeted antioxidant SkQ1 and partial inhibition by electron-leak suppressors S1QEL and S3QEL. Evidently, the insufficient H_2_O_2_ signal disabled BCKA-stimulated insulin secretion. A strict dependence of BCKA-stimulated insulin secretion on H_2_O_2_ at both phases in PIs was also evidenced by non-constant correlations of insulin release rates vs. H_2_O_2_ release rates, similarly to glucose (GSIS). Therefore, the presence or absence of this mt-PM H_2_O_2_ diffusion signal, rather than OXPHOS intensity alone, determines the ability of BCKAs to close K_ATP_ channels, initiate Ca^2+^ oscillations, and elicit insulin secretion.

For KIC, stoichiometric coupling between the H_2_O_2_ and insulin release roughly matches that one observed for GSIS. This supports a shared triggering mechanism on the plasma membrane of pancreatic β-cells, despite NOX4 being the H_2_O_2_ source with glucose [14]. Distinct metabolite routes for KIV and KMV result in different insulin-to-H_2_O_2_ stoichiometries. Together, these findings define a unifying mt-PM H_2_O_2_/redox-triggering mechanism by which BCKA metabolism engages β-cell stimulus-secretion coupling and refine the conceptual link between BCAA metabolism, mitochondrial redox homeostasis, and β-cell function.

This study identifies ETFQOR-driven mitochondrial H_2_O_2_ signaling as a dominant central mechanism linking β-like oxidation of KIC, KIV, and KMV to insulin secretion in murine pancreatic islets and INS-1E β-cells. An excessive QH_2_ input from ETFQOR induces superoxide formation at sites I_Q_ and III_Qo_ of Complex I and III, respectively. The secondary electron entries occur by regular routes of NADH to Complex I and succinate to SDH/Complex II (fed partially also by OXCT1). However, these inputs contribute by at most 20% to the overall superoxide formation. Employing specific sites I_Q_ and III_Qo_ electron leak suppressors [3,4], we demonstrated that the excessive QH_2_ input by ETFQOR is bifurcated (Fig. 4A), causing *i*) elevated superoxide formation at site I_Q_, due to the reversed electron transfer, i.e.*,* an effective product (QH_2_) inhibition of Complex I output of QH_2_; as well as, *ii*) elevated superoxide formation at site III_Qo_, due to the excessive incoming QH_2_.

Knowing that SkQ1H_2_ suppresses electron leaks at both these sites I_Q_ and III_Qo_ and resulting SkQ1 regenerates at site I_F_ [29], one would expect a higher inhibition of H_2_O_2_ flux with SkQ1 relative to sole S1QEL and sole S3QEL inhibition. Our data confirmed this, besides the extreme represented by no S3QEL inhibition of *J*^ext^ rates induced by KIV. Such apparent lack of site III_Qo_ contribution to superoxide formation in the case of KIV is explained by the highest NADH/NAD^+^ ratio created upon KIV β-like oxidation (3 NADH molecules plus those NADH of Krebs cycle created). Acting at the highest NADH/NAD^+^ ratio, a surplus of electron flow to the site III_Qo_ is not attenuated with S3QEL, since the site III_Qo_ contribution to the overall superoxide formation is minimum. Moreover, S3QEL acts downstream of electron transfer retardation in front of the Q cycle.

For KIC and KMV the surplus ETFQOR donation of QH_2_ also increases superoxide formation at site III_Qo_, as reflected by the partial S3QEL antioxidant action, suppressing electron leak at site III_Qo_. This results in an adequate partial inhibition of *J*^ext^ rates (Fig. 4A and B). A similar partial inhibitory effect of individual S1QEL or S3QEL stems from the release of the retardation of electron transfer at one site individually, and not at the other site. A little additive effect of S1QEL + S3QEL can be explained by switching to an alternative superoxide formation at site I_F_, increasing when the I_Q_ superoxide is prevented by redirection of the reverse electron transfer to S1QEL. Also, an alternative superoxide may also be formed within the ETFQOR protein itself, hypothetically at the site E_F_ [3].

The flavin site of superoxide formation, site I_F_, forms more superoxide at higher NADH/NAD^+^ ratio [3,15]. Hence, BDH silencing, which increased this ratio, increased the observed *J*_ext_ rates. Not counting the regular Krebs cycle, theoretically, the NADH yield decreases in order of KIV > KMV > KIC, while for KIC the yield could be zero, if all acetoacetate was converted to 3-hydroxybutyrate by BDH (Fig. 1). Experimental results confirmed these predictions (Fig. 2A; Fig. 4E).

For KIC and KMV, the third and again minor input regarding superoxide formation is acetyl-CoA, driving the Krebs cycle. The input of excessive succinyl-CoA after its conversion to succinate via Complex II/SDH [3,22] might also contribute for KIV and KMV. This is more utilized for KMV, having both inputs into the Krebs cycle, via acetyl-CoA and succinyl-CoA. The latter may contribute at a certain Complex II/SDH site by a little of additional superoxide. Alternatively, an additional superoxide fraction may result from the excessive QH_2_ input by Complex II/SDH, followed by the reverse electron transfer to site I_Q_. This process is predominant with KIV, as explained above. Note also, that the matrix BCKDH complex was itself suggested to produce superoxide [15].

We still did not explain why the yield of total insulin was maximum for KIC, lower for KIV, and minimum for KMV, despite attempting to double KMV dose to adjust the correct l-active form, since KMV was racemic. As respiratory studies have indicated, this could be due to the estimated OXPHOS intensity relative to maximum respiration capacity decreasing just in order of KIC > KIV > KMV (Fig. 7L). Such a slight decrease could be explained by more complex reactions for KIV and KMV, involving more enzymes and thus being slower. This is also reflected by the existence of lag phases in Ca^2+^ oscillations, showing that re-establishment of threshold membrane potential from which Ca_V_ channels are open requires a longer time to accumulate ATP and H_2_O_2_ and this causes the lag phase.

Previously, β-like oxidation was indicated by ^13^C-KIC incorporation into HMG-CoA [30]. Now we definitively demonstrate the existence of this pathway in INS-1E cells by unbiased MS metabolomics and by ^13^C-tracking. The latter concerned not only with Krebs cycle metabolites but also showed ^13^C incorporation into R-carnitines synthesized from the respective R–CoAs by carnitine-O-acetyltransferase (CRAT) [26]. CRAT has a substantial activity in INS-1E cells. For KIV we also showed that the ALDH2-ACSF3-MMUT side pathway has a negligible contribution in INS-1E cells.

Our experiments at higher BCKA concentrations likely induced a maximum H_2_O_2_ yield for the mt-PM H_2_O_2_ signaling when compared to the potentially existing physiological one. However, all inhibitory studies provided the identical mechanistic basis for the same interpretation also at 0.3 mM BCKAs. BCKAs are present in the plasma at 300 to 500 μM [31] and are translocated into the cytosol of PI β-cells via specific transporters [32] and to the mt matrix via the SLC16A1/MCT1 (monocarboxylate transporter 1) [31,32]. As our dose-responses and studies with antioxidants in PIs demonstrated, these physiological BCKA concentrations are sufficient for both mt-PM redox signals as well as modest insulin secretion. In mice, we have simulated KIC-stimulated insulin secretion and showed its time course to be similar to that observed for glucose (GSIS). We also showed that at physiologically relevant 0.25 mM KIC, amounts of insulin secretion given by AUCs reach about 30% of those for glucose (GSIS). It remains to be investigated whether there exists (if any) a contribution of blood/plasma BCAAs (Leu, Val, and Ile) to BCKA-SIS. Theoretically, this depends on the expression of BCAA transporters in β-cells [33,34].

## 5 Conclusions

This study identifies ETFQOR-driven mitochondrial H_2_O_2_ signaling as a central mechanism linking β-like oxidation of KIC, KIV, and KMV to insulin secretion in murine pancreatic islets and INS-1E β-cells. The surplus electron entry via ETFQOR into the respiratory chain generates superoxide and H_2_O_2_, which diffuses to the cell surface. The resulting mitochondria-to-plasma-membrane H_2_O_2_ signal—rather than OXPHOS alone—contributes to K_ATP_ channel closure and thus induces Ca^2+^ oscillations and resulting insulin exocytosis. The close similarity of insulin/H_2_O_2_ stoichiometries between BCKA-stimulated and glucose-stimulated insulin secretion supports a shared redox-triggering principle at the plasma membrane of β-cells. This extends the emerging concept that physiological ROS/H_2_O_2_ act as obligatory second messengers in nutrient-stimulated insulin secretion.

## CRediT authorship contribution statement

**Eduardo Klöppel:** Formal analysis, Investigation, Methodology. **Hana Engstová:** Data curation, Formal analysis, Investigation, Methodology. **Pavla Duspiva:** Investigation. **Jan Tauber:** Investigation, Resources. **Oleksandra Mozheitova:** Investigation, Methodology, Resources. **Martin Jabůrek:** Conceptualization, Data curation, Formal analysis, Investigation, Methodology, Validation, Writing – review & editing. **Petr Ježek:** Conceptualization, Data curation, Formal analysis, Funding acquisition, Methodology, Resources, Supervision, Validation, Writing – original draft, Writing – review & editing.

## Declaration of competing interest

There are no competing financial interests.

## Data Availability

Data will be made available on request.
