# Supplementary material for: Mitochondria-to-plasma-membrane H2O2 signaling induced by branched-chain keto acid oxidation essentially stimulates insulin secretion in pancreatic β-cells
Source: Redox Biol. 2026 Jul 16;96:104309. doi: 10.1016/j.redox.2026.104309 (PMC13452360; doi:10.1016/j.redox.2026.104309)
Supplement: Supplementary file 1 [file mmc1.pdf]

Supplementary material

for Redox Biology article

Mitochondria-to-plasma-membrane H_2O_2 signaling induced by branched-chain keto acid oxidation essentially stimulates insulin secretion in pancreatic β-cells

Eduardo Klöppel[1], Hana Engstová[1], Pavla Duspiva[1], Jan Tauber[1], Oleksandra Mozheitova[1], Martin Jabůrek[1], and Petr Ježek[1,2]

[1]Department of Mitochondrial Physiology, No.75, Institute of Physiology of the Czech Academy of Sciences, Vídeňská 1083, Prague, 14220, Czech Republic

[2]**Corresponding author:** Dr. Petr Ježek, PhD., DSc.

Dept. of Mitochondrial Physiology, No.75,

Institute of Physiology of the Czech Academy of Sciences,

Vídeňská 1083,

14220 Prague 4, Czech Republic

Tel.: 011-420-296442760

Fax: 011-420-296442488

E-mail: jezek@biomed.cas.cz

Keywords: *pancreatic β-cells / branched-chain keto acids / insulin secretion / mitochondrial H_2O_2 signaling / electron-transfer flavoprotein ubiquinone oxidoreductase*

Running head: ***Mitochondrial H_2O_2 signal in insulin secretion***

Part I BCKA metabolism

Recapitulating BCKA metabolism (Fig. 1; Fig. S1A), we note that the end products of **KIC β-like oxidation** are *acetoacetate* and *acetyl-CoA* [1,2]. Matrix BCKA-dehydrogenase (BCKDH) complex, which itself was suggested to produce superoxide [12], provides oxidative decarboxylation of KIC to isovaleryl-CoA, driven by CoA and NAD^+, with an $FAD^+/FADH_2$ catalysis, thus yielding the first NADH molecule. Isovaleryl-CoA is converted by isovaleryl-CoA dehydrogenase (IVD) to 3-methylcrotonyl-CoA, upon parallel FAD reduction to $FADH_2$. Next, methylcrotonyl-CoA carboxylase (MCCC) with bicarbonate and ATP provides 3-methylglutaroyl-CoA. This is converted by methyl-glutaryl-CoA hydratase (AUH) to 3-hydroxy-3-methylglutaryl-CoA, which is split by 3-hydroxy-3-methylglutaryl-CoA lyase (HMGCL) to acetoacetate and acetyl-CoA. Note that in overall KIC metabolism 3 NADH are yielded within the Krebs cycle (in the absence of citrate export and concomitant truncation after citrate synthase) plus one NADH is made by BCKDH. Hence, altogether maximum 4 NADH molecules could be released per cycle, plus $FADH_2$, plus acetoacetate.

However, acetoacetate might be captured either *i*) by β-hydroxybutyrate dehydrogenase (BDH/β-OHBDH; *aka* 3OHBDH) together with NADH, thus being converted to β-hydroxybutyrate (3-hydroxybutyrate) and NAD^+; or alternatively, *ii*) acetoacetate together with succinyl-CoA is transformed to acetyl-CoA by 3-oxoacid-CoA-transferase-1 (OXCT1/SCOT1, *aka* succinyl-CoA-3-oxaloacid-CoA transferase); or *iii*) it may penetrate to the cytosol, e.g. for fatty acid synthesis. Thus, **the net yield of $FADH_2$ and 4 NADH** (3 from the complete Krebs cycle) **results from KIC oxidation, if all acetoacetate penetrates to the cytosol.** The NADH yield remains at 4, when all acetoacetate is split with succinyl-CoA into acetyl-CoA (since succinyl-CoA, which would provide a single Krebs cycle, is withdrawn and this single cycle is then restored by Acetyl-CoA). Finally, if the BDH reaction favors *3-hydroxybutyrate formation* from acetoacetate, ***only 3 NADH*** are theoretically yielded. Note, the excess over glucose oxidation is zero NADH in this case; or a single NADH when 100% acetoacetate would penetrate the cytosol.

Importantly, IVD, yielding $FADH_2$, donates electrons via electron-transferring flavoproteins (ETF) to the inner membrane enzyme, ETF:coenzyme-Q oxidoreductase (ETFQOR). ETFQOR itself could produce superoxide, as suggested for skeletal muscle [18], but its major reaction product is the same as the product of Complex I. This is reduced Coenzyme Q, ubiquinol (QH_2). ETFQOR thus reduces ubiquinone (Q) to QH_2, which is coupled to oxidation of $FADH_2$ to FAD (though two ETF molecules are required for two transfer steps of single electrons to ETFQOR).

Upon **β-like oxidation of KIV** (Fig. 1; Fig. S1B), KIV is converted by the BCKDH complex to isobutyryl-CoA (yielding the first NADH), which is further processed by acyl-CoA dehydrogenase 8 (ACAD8, yielding $FADH_2$) to methylcrylyl-CoA (*aka* methacrylyl-CoA). The latter is hydrated by enoyl-CoA hydratase, short chain 1 (ECHS1/HADHA) to 3-hydroxyisobutyryl-CoA, while next 3-hydroxyisobutyryl-CoA hydrolase (HIBCH) reaction leads to 3-hydroxyisobutyrate. Next, 3-hydroxyisobutyrate dehydrogenase (HIBADH) provides methylmalonate semialdehyde at the NAD^+ expense, yielding the second NADH. Methylmalonate semialdehyde is transformed by a series of enzymatic reactions beginning with methylmalonate semialdehyde dehydrogenase (ALDH6A1) at the NAD^+ expense, yielding the third NADH and CO_2 plus propionyl-CoA. Its conversion by propionyl-CoA carboxylase PCCB, methylmalonyl-CoA racemase, and finally by methylmalonyl-CoA mutase (MUT) leads to succinyl-CoA. In conclusion, **$FADH_2$ and total 6 NADH may result from KIV oxidation** (3 from the complete Krebs cycle). However, the succinyl-CoA input should not exceed the "regular" Krebs cycle turnover here, since no additional acetyl-CoA input exists.

Finally, **β-like oxidation of KMV** (Fig. 1), starts again at the BCKDH complex, which converts KMV to 2-methylbutyryl-CoA (yielding the first NADH), which is processed by 2-methylbutyryl-CoA dehydrogenase (*aka* short/branched-chain-acyl-CoA dehydrogenase, SBCAD/ACADSB), yielding $FADH_2$, to tiglyl-CoA (2-methylcrotonyl-CoA). The latter is processed by enoyl-CoA-hydratase ECHS1/HADHA to 2-methyl-3-hydroxybutyryl-CoA without requiring ATP or bicarbonate; and next by 2-methyl-3-hydroxybutyryl-CoA dehydrogenase (HSD17B10) to 2-methylacetoacetyl-CoA at the NAD^+ expense, yielding the second NADH. Next, the 2-methylacetoacetyl-CoA thiolase (ACAT1) with CoA splits 2-methylacetoacetyl-CoA to acetyl-CoA and propionyl-CoA. Propionyl-CoA is converted by propionyl-CoA carboxylase (PCCB), methylmalonyl-CoA racemase (MCEE), and methylmalonyl-CoA mutase (MMUT) to succinyl-CoA. However, the carboxylase reaction requires again bicarbonate and ATP. The expected yield is **$FADH_2$** plus 2 NADH and 3 NADH within the complete Krebs cycle per acetyl-CoA, hence total 5 NADH, when **not counting the additional succinyl-CoA input into the Krebs cycle**. If this is counted as another one cycle, total **8 NADH** could be predicted for KMV oxidation plus **$FADH_2$**. The surplus of succinyl-CoA might also affect CII/SDH reaction and could induce increased superoxide formation therein.

All the above metabolic pathways occur superimposed on low-glucose metabolism in β-cells with the steady state yield of 3 NADH per cycle in the absence of pyruvate-based redox shuttles [9,10,23]. Hence, the onset of KIC,

KIV, and KMV oxidation brings instead 3–4, 6 and 8 NADH per cycle, respectively. This is expected to cause a sufficient NADH pressure (NADH/NAD^+-ratio) to induce excessive superoxide formation at the Complex I site I_F. Nevertheless, ETFQOR supply of the surplus of QH_2, may induce reverse electron transfer further affecting redox homeostasis as documented in the main article. Moreover, with KIV and KMV excessive fluxes involving Complex II might also induce superoxide formation.

Analysis of the **KIC β-like oxidation pathway with regard to ^{13}C tracing** is then illustrated in the scheme of Fig. S1A. Note, that from the original M+6 U-^{13}C-KIC, the M+5 intermediates are formed after the BCKDH reaction until 3-hydroxy-3-methylglutaryl-CoA lyase (HMGCL) splits the last intermediate (3-hydroxy-3-methylglutaryl-CoA) to M+2 acetyl-CoA and M+3 acetoacetate [25]. If 3-hydroxybutyrate is formed, its M+3 isomer should be found when it is accumulating. The OXCT1 reaction of M+3 acetoacetate with M+0 (or M+2) succinyl-CoA provides M+2 acetyl-CoA and M+0 (or M+2) succinate, entering back the Krebs cycle.

Similarly, analysis of the **KIV β-like oxidation pathway with regard to ^{13}C tracing** is shown in the scheme of Fig. S1B. Note, that from the original M+5 U-^{13}C-KIV, the M+4 intermediates, such as isobutyryl-CoA, exist after the BCKDH reaction until the 3-hydroxyisobutyrate dehydrogenase (HIBADH) reaction product methylmalonate semialdehyde. Next, if branching of KIV oxidation proceeds, yielding would be different, and either M+3 or M+4 derivatives would be formed. When aldehyde dehydrogenase-2 reaction proceeds, M+4 methylmalonate semialdehyde is simply oxidized to carboxylic acid, i.e., methylmalonate. This could be followed by the ACSF3 reaction (acyl-CoA synthetase family member 3 reaction), forming M+4 R-methylmalonate-CoA. Next, methylmalonyl-CoA mutase (MMUT) will mutate it to M+4 succinyl-CoA, entering the Krebs cycle. However, when methylmalonate-semialdehyde dehydrogenase [acylating], mitochondrial, i.e., aldehyde dehydrogenase 6 family, member A1 (ALDH6A1) reaction takes place, it loses a single carbon in the form of CO_2 and generates NADH, M+4 methylmalonate semialdehyde is converted to M+3 propionyl-CoA. Despite this is followed by carboxylation provided by propionyl-CoA carboxylase (PCCB), ^{12}C-CO_2 enters this reaction, hence M+3 S-methylmalonyl-CoA is the reaction product, which is further isomerized to M+3 R-methylmalonyl-CoA by methylmalonyl-CoA epimerase (MCEE) and further to M+3 succinyl-CoA by MMUT. Since M+3 succinyl-CoA also enters to the Krebs cycle, one can determine ratios of M+4 to M+3 derivatives, which reflects the respective contribution of the two pathways.

In all schemes, we can also assume that all formed R-CoA derivatives can be transformed into the corresponding R-carnitines by carnitine O-acetyltransferase (CRAT; also called carnitine acetyltransferase, CAT; [26]). Since MS detection of R-carnitines is much easier than for R-CoAs, we are using R-carnitines as surrogates for R-CoAs. Of course, only a small fraction of R-CoA intermediates could be converted, so this way requires very high sensitive detection. To overcome this, we also supplied the assay by 1 mM L-carnitine.

It remains to be investigated whether there exists (if any) a contribution of blood/plasma BCAAs (Leu, Val, and Ile) to BCKA-SIS. Theoretically, this depends on the expression of BCAA transporters in β-cells, such as heterodimeric LAT1 composed of SLC7A5 and SLC3A2, which is glycoprotein CD98 ensuring the exchange of BCAA import for glutamine export [33]. Also, the Na^+ symporter SLC16A19 imports BCAAs into the cell cytosol [32]. In brown adipose tissue, mitochondrial carrier SLC25A44 imports BCAAs into the mitochondrial matrix [34]. The LAT1 BCAA import is not compatible with a parallel glutaminolysis, directing rather glutamine uptake into cells. Note also, there is no BCKDH in the cytosol and β-cells also do not express cytosolic BCAT1 [1]. Oxidation of BCAAs is therefore dependent on their mitochondrial uptake, provided by the SLC25A44 carrier, which could be much slower relative to SLC16A1/MCT1 providing BCKA uptake.

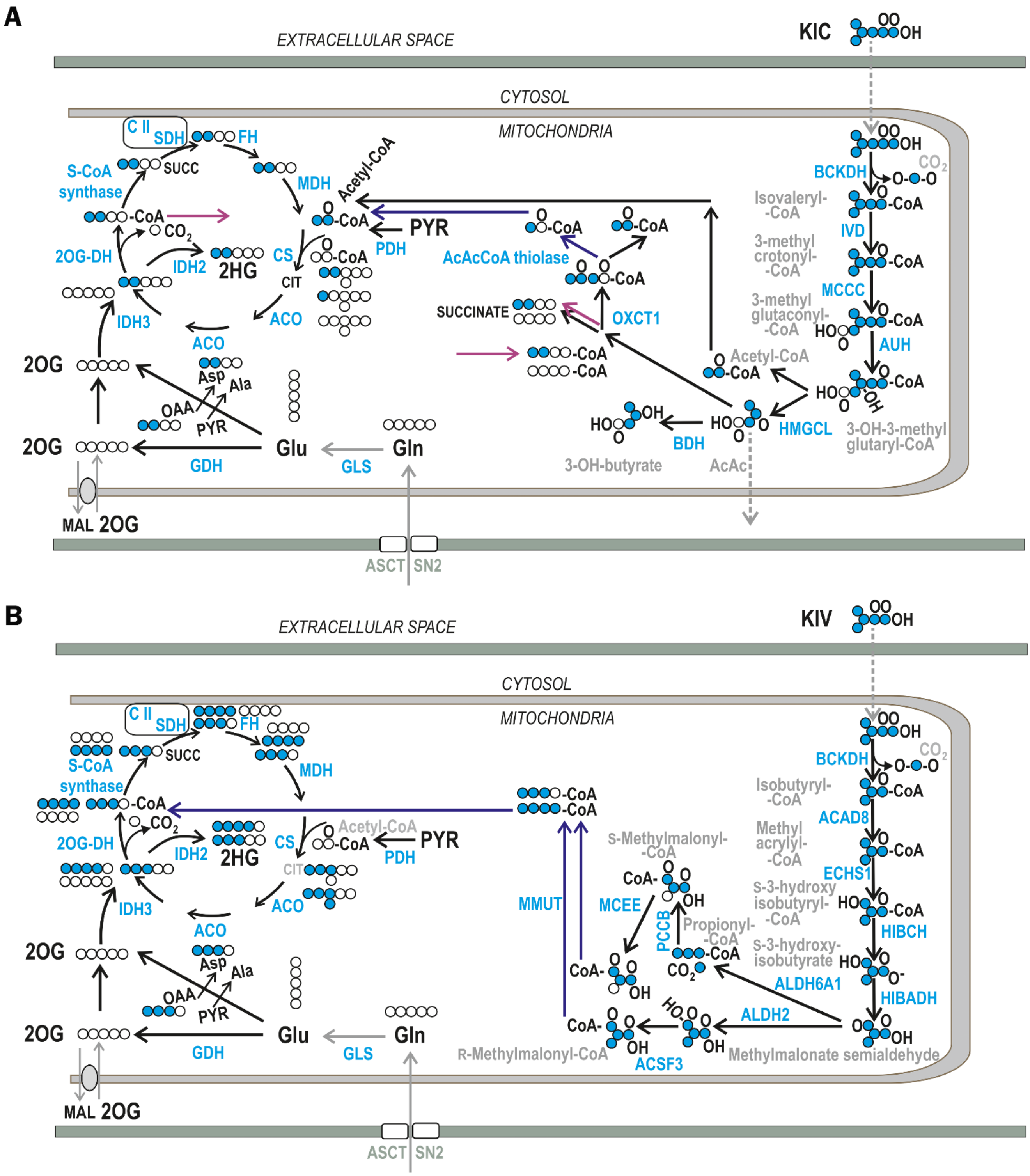

Figure S1. A) **KIC metabolism with emphasized number of ^{13}C carbons introduced from U-^{13}C-KIC** – *Blue* atoms represent ^{13}C carbon. Reaction products of KIC β-like oxidation are M+2 acetyl-CoA, entering the Krebs cycle, plus M+3 acetoacetate (AcAc). M+2 acetyl-CoA provides the M+2 Krebs cycle metabolites. The M+3 acetoacetate can either penetrate to the cytosol or may enter to the OXCT1 reaction or may be converted by BDH to M+3 3-hydroxybutyrate („3-OH-butyrate“). The OXCT1 reaction, consuming M+3 AcAc plus M+2 (*magenta*) or M+0 succinyl-CoA (S-CoA), provides an alternative turn of the Krebs cycle with the input of M+1 acetyl-CoA (*dark violet*), thus generating also M+1 metabolites of the Krebs cycle (for simplicity, only citrate is emphasized). *Abbreviations absent in the main text*: 2OG-DH – 2-Oxoglutarate dehydrogenase; AcAc – Acetoacetate; ACO – Aconitase; ASCT – Neutral amino acid transporter, termed also SLC1A5; AUH – 3-Methylglutaconyl-CoA hydratase; CS – Citrate synthase; FH – Fumarate hydratase; GDH – Glutamate dehydrogenase; GLS – Phosphate-activated mitochondrial glutaminase; HMGCL – 3-Hydroxy-3-methylglutaryl-CoA lyase; IDH2,3 – Isocitrate dehydrogenase 2, 3; IVD – Isovaleryl-CoA dehydrogenase; MDH – Malate dehydrogenase 2 ; MCCC – Methylcrotonyl-CoA carboxylase; PDH – Pyruvate dehydrogenase; PYR – Pyruvate; SN2 – System N2 transporter.

B) **KIV metabolism with emphasized number of ^{13}C carbons introduced from U-^{13}C-KIV** – *Blue* atoms represent ^{13}C carbon. Reaction products of KIV β-like oxidation are M+3 and M+4 succinyl-CoA, entering the Krebs cycle. *Abbreviations absent in the main text and panel A*: ACSF3 – acyl-CoA synthetase family member 3 reaction; ALDH6A1 – aldehyde dehydrogenase 6 family, member A1, *i.e.* methylmalonate-semialdehyde dehydrogenase [acylating], mitochondrial; HIBADH – 3-hydroxyisobutyrate dehydrogenase; MCEE – methylmalonyl-CoA epimerase; MMUT – methylmalonyl-CoA mutase; PCCB – propionyl-CoA carboxylase.

Part II BCKA–induced superoxide/H_2O_2 formation and consequences for insulin secretion

II.1. Saturated rates of extracellular H_2O_2 release induced by BCKAs –Amplex UltraRed monitoring in INS-1E cells First, we show saturated rates measured at 10 min after addition of 1 mM BCKAs (Fig. S2A) and dose responses for these saturated rates. (Fig. S2B). Remaining activity in ETFQOR-silenced cells for initial rates with 1 mM BCKAs (Fig. S2C) and saturated rates with 1 mM BCKAs (Fig. S2D) and 7.5 mM BCKAs (Fig. S2E) is then displayed. While reaching maximum reaction later at 1 mM BCKAs, the inhibitory ability of ETFQOR siRNA was lower. Control western blots are shown to judge the effects of silencing (Fig. S2F). SkQ1-sensitive rates are shown for MitoSOX assays (Fig. S2G).

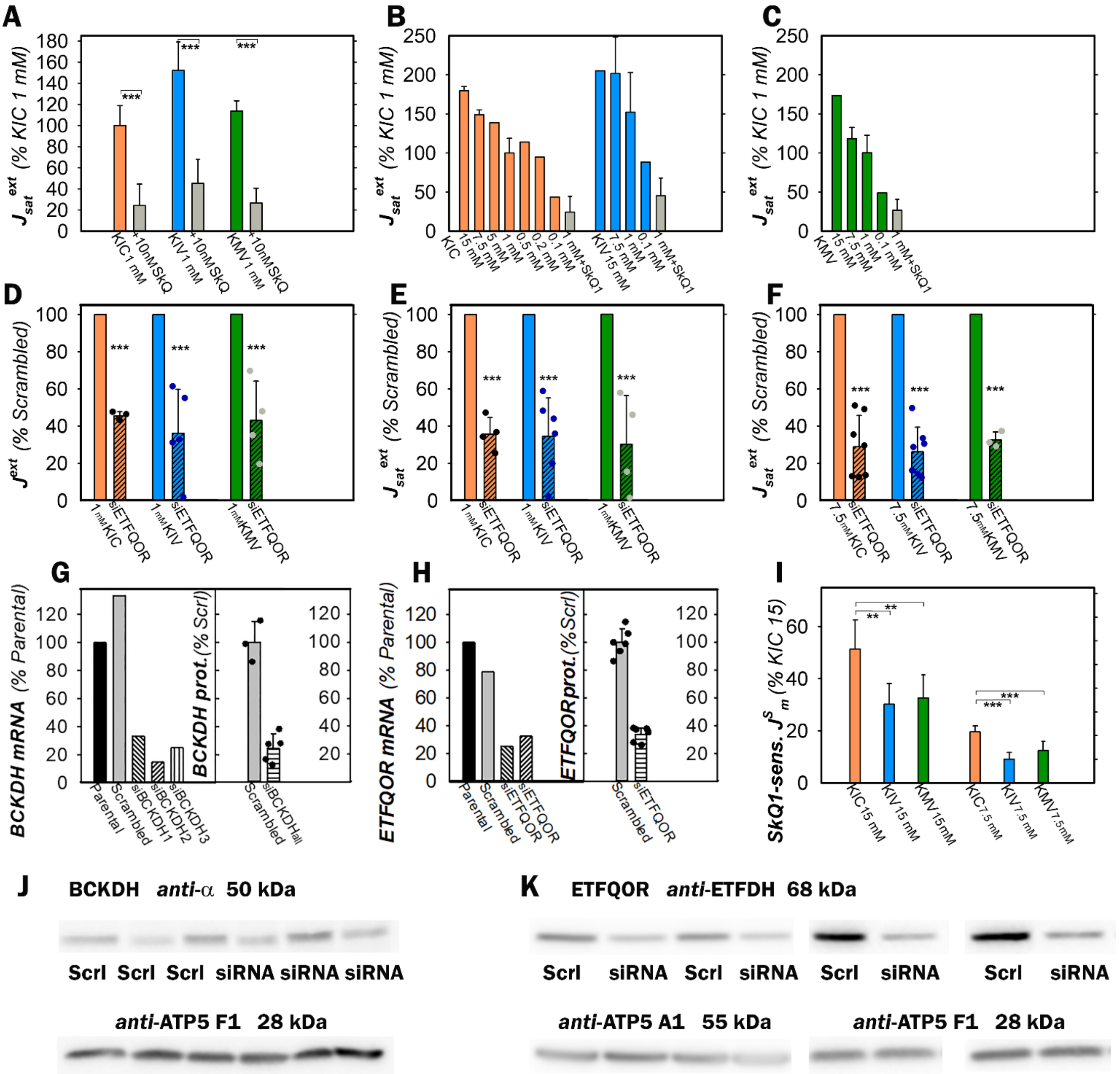

Figure S2. Rates of the extracellular H_2O_2 release monitored with Amplex UltraRed in INS-1E cells and strength of BCKDH and ETFQOR silencing – A–C) Dose-responses for saturated rates at the given doses of KIC, KIV and KMV without and with 10 nM SkQ1. **D–F**) Remaining activity in ETFQOR-silenced cells for initial rates with 1 mM BCKAs (**C**) and saturated rates with 1 mM BCKAs (**D**) and 7.5 mM BCKAs (Fig.**E**) Panels **G**,**H)** shows **PCR detection** of the corresponding transcripts and residual protein content after silencing derived from **western blots** (exemplar ones shown in panels **J**,**K**) to survey effects of BCKDH and ETFQOR silencing. **I)** SkQ1-sensitive rates are summarized for MitoSOX assays. Data was collected from N biological replicates (cell passages) and n time courses as follows: A–C) *N*=2, *n*=3 for KIC 15 mM; *N*=3, *n*=3 for KIC 7.5 mM; *N*=10, *n*=15 for KIC 1 mM; and *N*=4, *n*=8 for KIC 1 mM plus 10 nM SkQ1; similarly, *N*=2, *n*=3 for KIV 15 mM; *N*=3, *n*=7 for KIV 7.5 mM; *N*=5, *n*=11 for KIV 1 mM; and *N*=5, *n*=7 for KIV 1 mM plus 10 nM SkQ1; and *N*=3, *n*=3 for KIV 15 mM; *N*=3, *n*=4 for KIV 7.5 mM; *N*=7, *n*=12 for KIV 1 mM; and *N*=5, *n*=6 for KIV 1 mM plus 10 nM SkQ1. After ETFQOR siRNA treatment (*N*=3; and pairwise treatment with scrambled siRNA) *n*=3–4 for KIC, KIV, and KMV, respectively. For MitoSOX (**I**) *N*=3, cell passages; *n*=12, *n*=14 and *n*=12 were for, KIC, KIV and KMV, respectively.

II.2. Two-way factorial ANOVA for estimation of ETFQOR silencing effects on insulin secretion

For the KIC-stimulated insulin-release time-course experiment comparing between Scrl INS-1E cells (transfected by scrambled siRNA; Mission siRNA Universal Negative Control, Merck, SIC001) and cells silenced for ETFQOR (siETFQOR; i.e., ETFDH silencing Mission siRNAs, equal mix of Merc, SASI_Rn01_00096001 and SASI_Rn01_00096002), insulin secretion rates were first calculated for each independent replicate as slopes of accumulated insulin release over time. These rates were analyzed by two-way factorial ANOVA with stimulation (considering rates with no addition vs. rates with KIC addition in Scrl cells) and ETFQOR siRNA treatment (no addition siETFQOR vs. siETFQOR) as fixed factors, followed by Holm-Sidak post hoc tests of simple effects. The interaction term stimulation vs. siETFQOR was used to test whether ETFQOR silencing specifically altered the KIC-induced component of insulin secretion.

This analysis showed a significant interaction between stimulation and ETFQOR silencing which was characterized by ($F(1,8) = 14.73$; $P = 0.00496$), indicating that ETFQOR silencing selectively suppressed the KIC-induced secretion rate. KIC addition significantly increased differential insulin secretion rate by 2.12 ± 0.38 ng.10^{-6} cells per min ($P = 0.0002$), whereas after ETFQOR silencing this was insignificant (rates increased by 0.33 ± 0.88 ng.10^{-6} cells per min; $P = 0.332$), accounting for 84% inhibition. Data for GSIS and ETFQOR silencing were treated analogously. Two-way factorial ANOVA yielded no significant interaction between glucose stimulation vs. siRNA ETFQOR ($F(1,8) = 1.177$; $P = 0.310$). Glucose addition significantly increased differential insulin secretion rate by 2.41 ± 0.39 ng.10^{-6} cells per min ($F(1,8) = 133$; $P = 3.10^{-6}$), which was similar after ETFQOR silencing (rates increased by 2.00 ± 0.60 ng.10^{-6} cells per min; $P = 0.332$), yielding non-significant 17% inhibition. Thus, unlike KIC-stimulated insulin secretion, GSIS was not significantly affected by ETFQOR silencing.

Detailed protocol for silencing of INS-1E cells and its validation

Cells were seeded at a density corresponding to approximately 60–70% confluency at the time of transfection. Prior to transfection, the culture medium was replaced with fresh complete growth medium 30–60 min in advance. The specific siRNAs for given gene were employed. Silencer Select technology (ThermoFisher Scientific, #4390771) was chosen with following IDs: BDH1 - s138562, OXCT1 - s234928, BCKDHA - s129255. For ETFDH silencing Mission siRNAs (Merck, SASI_Rn01_00096001, SASI_Rn01_00096002) were used as an equimolar mixture. For scrambled samples, Mission siRNA Universal Negative Control was used (Merck, SIC001). siRNA transfection complexes were prepared using GenMute™ transfection reagent according to the manufacturer's instructions. Briefly, siRNA was diluted in the transfection buffer to achieve a final siRNA concentration of 20-40 nM in total. After dilution of siRNA, GenMute™ transfection reagent was added at a constant reagent-to-siRNA ratio, and the mixture was gently mixed. The transfection complexes were incubated for 15 min at room temperature to allow complex formation and subsequently added dropwise to the cells. Cells were incubated with the transfection complexes overnight under standard culture conditions. Following incubation, the medium was replaced with fresh complete growth medium, and cells were further cultivated 48–72 hours until downstream analyses.

Silencing was verified by qPCR and Western blot. For qPCR, the Forget-Me-Not™ EvaGreen® qPCR Master Mix (Biotium) was used with a standard protocol. See below for primer sequences. The following antibodies were used for Western blots: ETFDH (Abcam, ab131376), OXCT1 (Cell Signaling, #92192), BCKDHA (Abcam, ab90691). qPCR primers were:

BCKDHA – Fw: ATCCCCATCTACCGCGTCA, Rev: GTCCCCTCCTCGCCATAGTT,

ETFDH – Fw: CCCGCCTCTCTCTGTTGTAT, Rev: CACTGATATGCAGGGCAGGA,

OXCT1 – Fw: ATTGGCTCGTTTGCTCCAGA, Rev: GGGCTTACCAGACTTGGCTT,

BDH1 – Fw: CTTGAGAAACAGAGGCAACGC, Rev: TGTGACAGGGGTCTGGAAAG

Normalization of qPCR according Pfaffl M.W., Nucl Acid Res 2001, 29(9):e45, was relative to the rat YWHAZ transcript, i.e., transcript of tyrosine 3-monooxygenase/tryptophan 5-monooxygenase activation protein zeta, 14-3-3 adapter protein; for which the primers were:

YWHAZ – Fw: AGCCCGTAGGTCATCTTGGA, Rev: TGCGAAGCATTGGGGATCAA

II.3. Ca^{2+} oscillations monitored with GCaMP fluorescence probe

Ca^{2+} oscillations were monitored with GCaMP fluorescence probe in transfected cells (Fig.3B–F) or in isolated PIs of heterozygous GCaMP6sβoe mice (Fig.3G–N; Fig.S3). The fluorescence of transfected islets was already recognized within the pancreas (Fig.S3A) and in isolated PIs (Fig.S3C) as compared to background (Fig.S3B). Ca^{2+} oscillations were recorded in isolated islets (e.g. Fig.S3D–I) separately for each cell which represented a single ROI (e.g. Fig.S3F,I). The numerical derivative was calculated to identify peaks of oscillations, so that a peak at time t_i occurred for the derivative of $F[Ca]_c(t_i)$ being zero at time t_i. Oscillations peaks in each trace were sorted by intensities into ranges scaled by 1/10 of maximum intensity (deciles or 10-percentiles) as summarized by histograms.

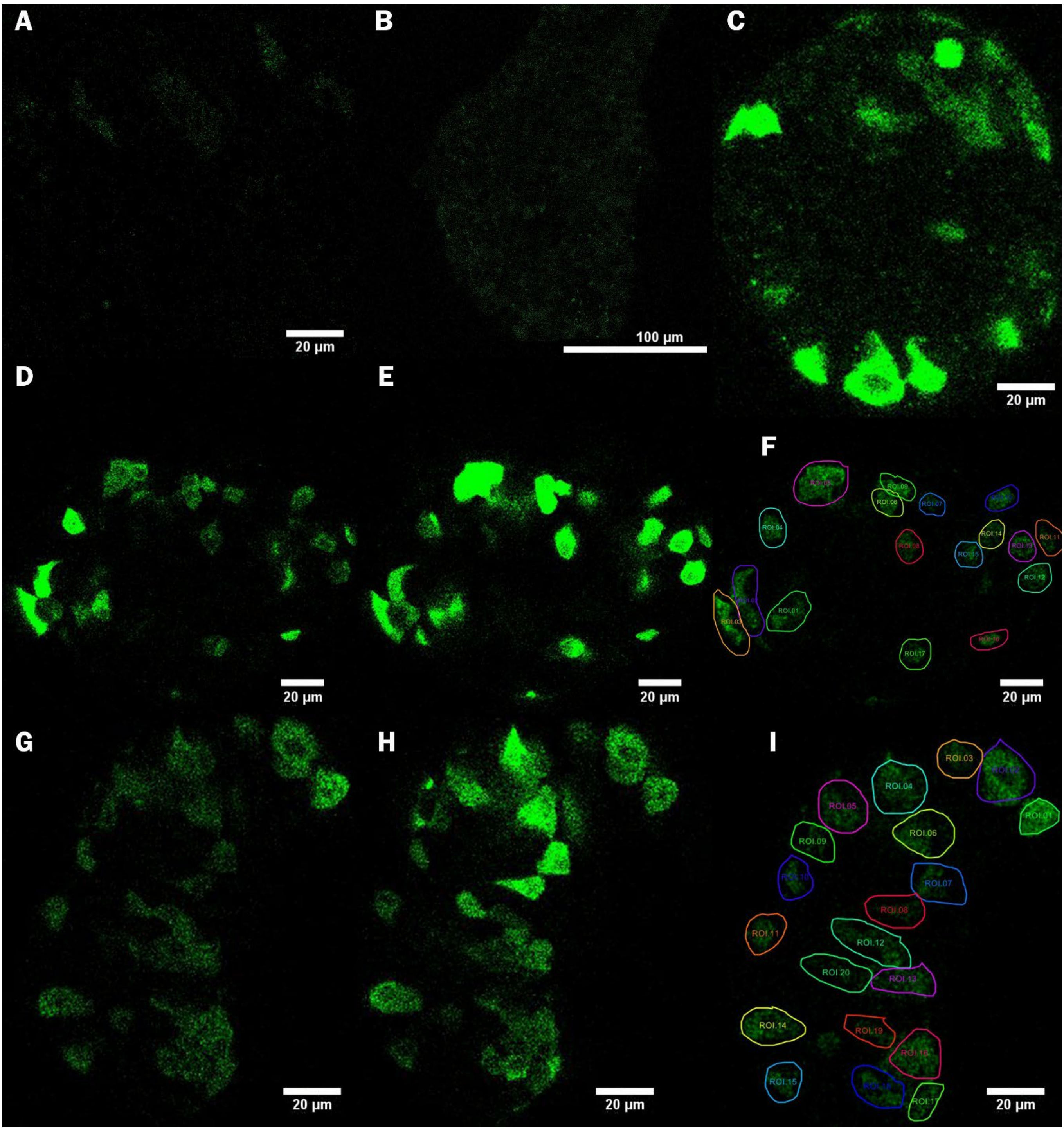

Figure S3. GCaMP fluorescence in the pancreas (A) and isolated PIs of heterozygous GCaMP6sβoe mice (C–I) – as compared in the corresponding confocal micrographs to background fluorescence of a single isolated PI (**B**). PIs were incubated with 5.5 mM glucose (**C**,**D**,**G**) to which 10 mM KIC was adjusted (**E**,**H**) and ROI corresponding to each single cell (**F**,**I**) were analyzed for Ca^{2+} oscillations intensities.

Detailed protocol for INS-1E cells

The vectors pGP-CMV-GCaMP6f (Plasmid #40753) and pGP-CMV-GCaMP6s (Plasmid #40755) were purchased from Addgene, which express the fast-responding and slow-responding but more sensitive GCaMP6 fluorescent Ca^{2+}indicator, respectively. GCaMP6 represents a synthetic fusion of green fluorescent protein (GFP), calmodulin and M13, a peptide sequence from myosin light-chain kinase. Cells were transfected using Lipofectamine™ 2000 (ThermoFisher) for 48 hours. A Leica TCS SP8 confocal microscope was employed for the time-lapsed recording of integral fluorescence intensity within the individual cells ROI with excitation at 480 and emission at 510 nm. For every second, integral fluorescence intensities $F[Ca]_c(t_i)$ were collected from the image of each responding single cell in widefield and data were plotted as a time course. Typically, 10 min periods (trails) were set and in each period the glucose level was adjusted, starting from 3 mM, then typically desired concentration of BCKA was added and at the end 30 mM KCl was added. Alternatively, 9 mM glucose was added and followed. Numerical 1st-derivatives were calculated to ascertain peaks of oscilations, so that a peak at time t_i occurred when the derivative of $F[Ca]_c(t_i)$ was zero at time t_i. Only those zero derivatives were selected when preceded by the positive derivative and followed by the negative derivative values, thus excluding the negative-positive derivative pairs around the zero derivative, which represent functional minima. Peaks of oscillations in each period were sorted by intensities into ranges scaled by 1/10 of maximum intensity (deciles) and histograms were plotted for each period.

Detailed protocol for pancreatic islets isolated from heterozygous GCaMP6sβoe mice

Murine pancreatic islets (PIs) from heterozygous GCaMP6s*β*oe mice were isolated in the same way as PIs from wt mice [11,14,23]. Typically, 5–15 isolated PIs were seeded to coverslips for 1 h and very carefully overlaid with a Krebs-Ringer HEPES (KRH) buffer (135 mM NaCl, 3.6 mM KCl, 10 mM HEPES, 0.5 mM MgCl2, 1.5 mM CaCl2, 0.5 NaH2PO4, 2 mM L-glutamine, 0.2% bovine serum albumin, BSA, pH 7.4), containing 5 mM glucose. Leica TCS SP8 confocal microscope was again employed for the time-lapsed recording of integral fluorescence intensity within the individual cells ROI with excitation at 480 and emission at 510 nm. Typically, up to four islets were observed per field. Histograms were calculated as described above for INS-1E cells.

II.4. Inhibitory ability for S1QEL, S3QEL and their combination and the respective saturated rates of extracellular H_2O_2 release induced by BCKAs – monitored by Amplex UltraRed + HRP in INS-1E cells

Suppressors of superoxide formation at sites I_Q (within the Complex I) and III_{Qo} (within the Complex III), i.e., S1QEL, S3QEL, respectively [15–20,23] were tested, individually and in combination to determine whether they inhibited the H_2O_2 release rates to the INS-1E cell exterior (J_{ext} rates) upon BCKA-SIS. Data for initial rates are contained in Fig.4A, whereas for saturated J_{sat}^{ext} rates (taken up to 10 min of records) are shown in Fig.S4A. Their re-calculations for the inhibitory ability concerning initial rates are shown in Fig.S4B and for saturated J_{sat}^{ext} rates in Fig.S4C. Fig. Fig.S4D then shows similar data for J_{sat}^{ext} rates and inhibition by atpenin.

II.5. Silencing of auxiliary enzymes for β-like oxidation of BCKAs as a tool for estimating relevance of these reactions – tests by Amplex UltraRed in INS-1E cells

Data showing existence of silencing of the enzymes studied BDH and OXCT1 are displayed in Fig.S4E–H. For protocol see part II.2. The major consequences of the absence of the respective enzymes on the metabolic fluxes relevant for superoxide formation in mitochondria is demonstrated in Fig.4E–G.

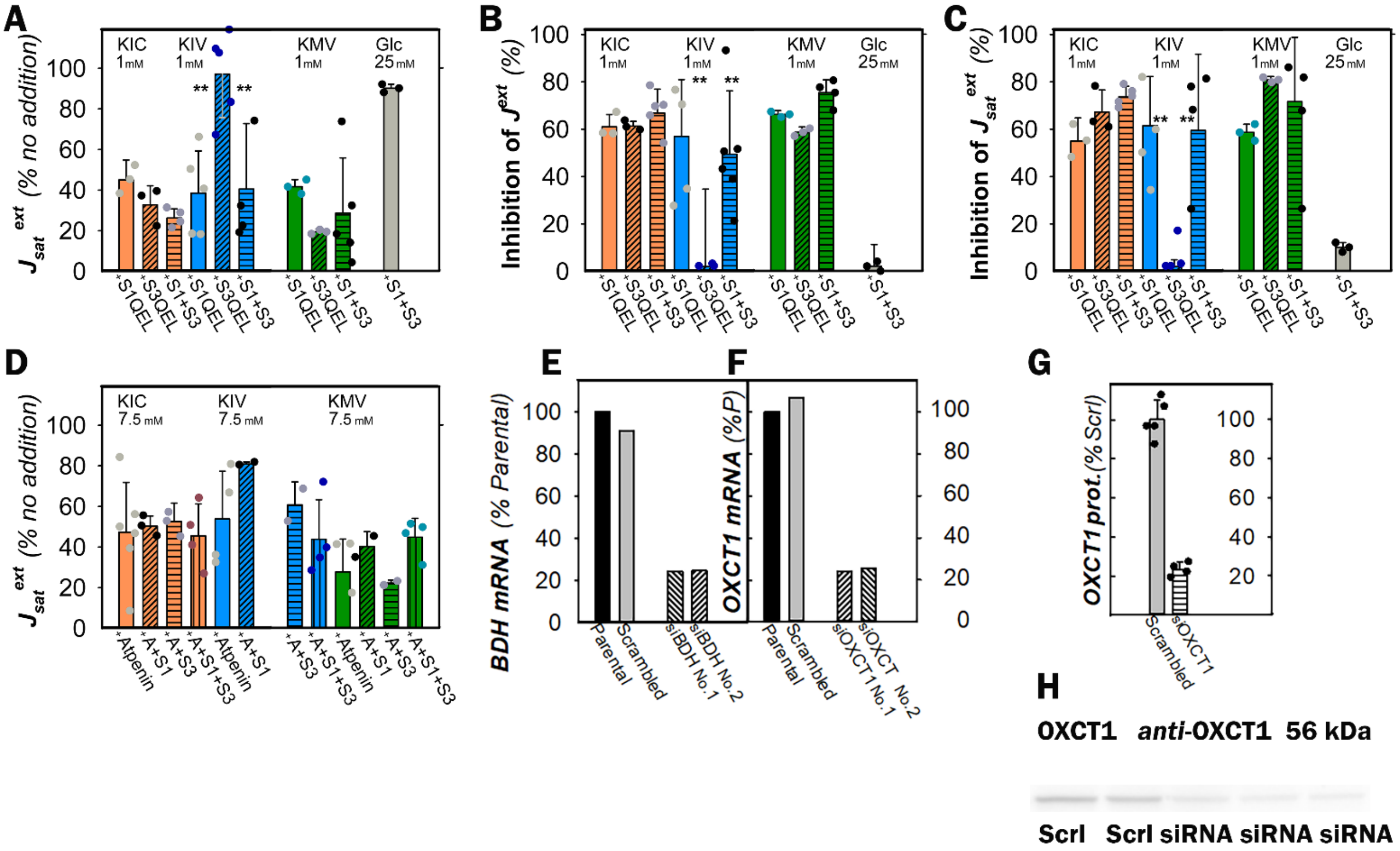

Figure S4. **Effects of S1QEL, S3QEL, and atpenin on H_2O_2 release induced by BCKAs and silencing of enzymes for auxiliary reactions of β-like oxidation – A) Inhibition of saturated J_{sat}^{ext} rates** by S1QEL, S3QEL (remaining activity). **B,C) Inhibitory ability** of S1QEL, S3QEL or both in combination for the initial rates (**B**) and saturated rates (**C**). (*N*=3–5, *n*=3–6). **D) Inhibition of saturated J_{sat}^{ext} rates** (remaining activity) by 0.5 μM atpenin. **E–H) Strength of silencing** as judged from **PCR detection** of the transcripts of BDH (**E**) and OXCT1 (**F**) and **residual protein content** for OXCT1 (**G**) evaluated by western blotting with the respective antibodies (**H**). Unreliable antibodies for BDH did not allow to screen BDH protein content.

II.6. Unbiased metabolomics and lipidomics analyzing BCKA β-like oxidation

Unbiased metabolomics showed also accumulation of 2-hydroxyglutarate (2-HG) upon KIV and KMV oxidation in INS-1E cells (Fig.5C,E). Concerning 2-hydroxyglutarate sources, one can predict that several enzymes may contribute to 2-HG formation under these conditions, which are permissive for the following reactions: *i*) incomplete isocitrate dehydrogenase-2 (IDH2) reaction consuming 2-OG and creating R-2-HG; *ii*) side malate dehydrogenase (MDH2) reaction, creating S-2-HG from 2-OG, which is also facilitated by the increased oxaloacetate (OAA) consumption at reversed MDH2 reaction. OAA is produced also by pyruvate carboxylase (PCarbox). Note that the observed fumarate and malate accumulation supports at least a partial reversal of MDH2, while reflecting also the additional succinyl-CoA (S-CoA) input. Finally, *iii*) phosphoglycerate dehydrogenase (PHGDH) could synthesize R-2-HG due to 2-OG accumulation.

Also, lipidomics was analyzed after 60 min incubations of INS-1E cells with KIC (Fig.S5A), KIV (Fig.S5B), and KMV (Fig.S5C); all at 15 mM and *N*=6. The MS-DIAL 4.92 software was used to analyze "lipidomics" with the following parameters: (*i*) data collection: MS1 tolerance, 0.01; MS2 tolerance, 0.025; (*ii*) peak detection: minimum peak height, 15,000; mass slice width, 0.05; smoothing method, Linear Weighted Moving Average; smoothing level, 3; (*iii*) MS/MS identification setting: accurate mass tolerance (MS1), 0.005; accurate mass tolerance (MS2), 0.005; identification score cut off, 80%; (*iv*) alignment: retention time tolerance, 0.05 min; MS1 tolerance, 0.01 Da; peak count filter, 5%; gap filling by compulsion, true. Complex lipids were annotated based on accurate MS1 and MS/MS spectra matching with in silico MS/MS spectra. For free fatty acids only accurate mass MS1 matching was used, considering elution order of fatty acids based on the number of carbons and double bond in the molecule. Data were filtered by removing lipids with a max sample peak height/blank peak height average < 0.8 from a dilution series (0, 1/16, 1/8, 1/4, 1/2, 1) of QC sample, and a relative standard deviation (RSD) > 30% from QC samples injected between 10 actual study samples.

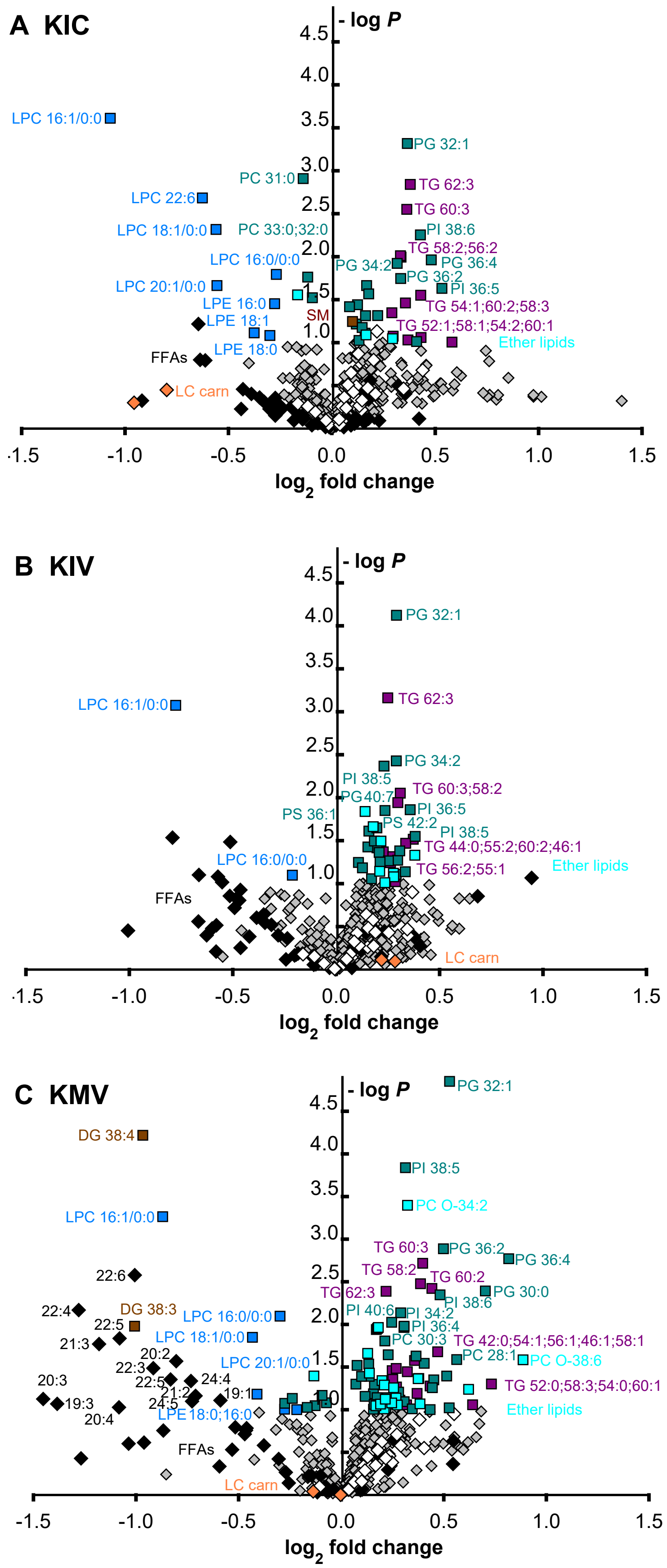

Figure S5. Volcano plots for lipidomics surveyed after 60 min of INS-1E cells incubation with KIC (A), KIV (B), or KMV (C), (all 15 mM).

II.7. Perifusion of pancreatic islets for assessing time course of insulin secretion and H_2O_2 release rates

Pancreatic islet perifusion with KIC and KIV and parallel survey of insulin and H_2O_2 in perifusates allowed us to correlate insulin secretion rates with rates of H_2O_2 release to the cell exterior. Such correlation for these assays is shown in Fig.8I, where linear or parabolic fits of all points or a fit of only data for the 1st phase (up to 14 min) were drawn. The meaning of these plots becomes clearer when PIs are challenged with antioxidants, such as SkQ1 (Fig.8J) or S1QEL (Fig.8K) and S3QEL (Fig.8M). The latter, however, does not act on KIV-stimulated insulin secretion in the 1st phase. To illustrate further results, also panels of Fig.S6 are shown with the corresponding fits of the 1st and 2nd phases separately.

As seen for KIC (Fig.S6A), an apparent stoichiometry can be obtained from the linear fits, despite rather non-linear correlation exists specifically for the 1st phase (Fig.8I). A steeper character of insulin vs. H_2O_2 release dependence is compatible with a threshold and triggering character of the essential redox signaling.

An inhibition by the S1QEL antioxidant has the 1st phase slope roughly equal to the 1st phase of the non-inhibited sample (Fig.S6B). This indicates that the remaining H_2O_2 release (originating from superoxide of the Complex III site III_{Qo}) induces the same insulin triggering mechanism. However, very low, highly inhibited, insulin secretion at H_2O_2 release which is roughly half-suppressed exists for the 2nd phase. Resulting insulin vs. H_2O_2 release dependence has therefore a very low slope (Fig.S6B). Similar insulin vs. H_2O_2 release dependence having a low slope, i.e. stoichiometry, was obtained for KIC and S3QEL antioxidant, acting at site III_{Qo} of the Complex III (Fig.S6C).

Analogous data are recognized for KIV, although represented by a lower burst in the 1st phase, ranking from zero to 0.06 ng insulin per pmol of H_2O_2 (fit yielded 0.027 ng/pmol) and with 2nd phase stoichiometry of 0.01 ng/pmol (Fig.S6D). These data showed no inhibition when S3QEL antioxidant was present (Fig.S6F) but reflected inhibition with S1QEL (Fig.S6E).

We can compare the above results with data of 0.3 mM BCKAs (Fig.9) and GSIS data (Fig.8O). For 7.5 mM glucose the 1st phase linear burst reached up to 0.028 pg insulin per pmol of H_2O_2, knowing that the H_2O_2 source is now the NOX4 enzyme. An average stoichiometry of the 1st phase (linear fit within Fig.8O) was 0.018 ng/pmol. The 2nd GSIS phase reached maximum stoichiometry of 0.010 ng/pmol with an average (linear fit within Fig.8O) of 0.0075 ng/pmol. Altogether, similar stoichiometries for GSIS and BCKA-IS data support the identical triggering principle existing on the plasma membrane of pancreatic β-cells.

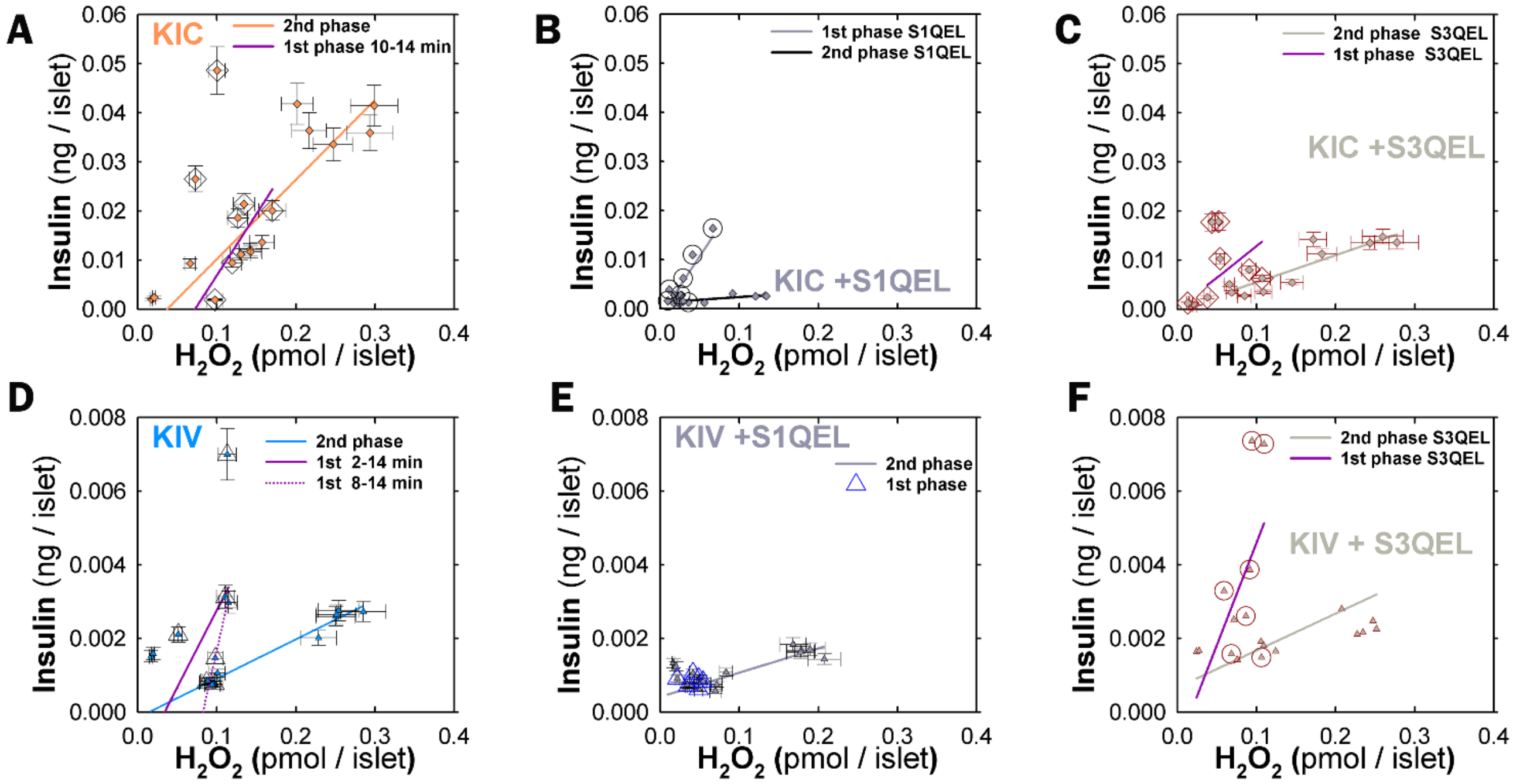

Figure S6. **Correlation of insulin secretion rates *vs.* rates of H_2O_2 release to the cell exterior** – Correlation of insulin secretion rates (from Fig.6, panels **H**,**I**) vs. corresponding rates of H_2O_2 release to the cell exterior Fig.8, panels **E**,**F**) were plotted for the 1st phase of insulin secretion (large *contours*) and 2nd phase (*without contours; see legend*). Separate fits for the 1st phase, 2nd phase are plotted in the graphs as indicated. KIC and KIV were 7.5 mM.

Pancreatic islet perifusion with 0.3 mM KIC and 0.3 mM KIV are shown in Fig.7A,B,D,E. Here we illustrate islet perifusion with 0.3 mM racemic KMV (Fig. S7A,B).

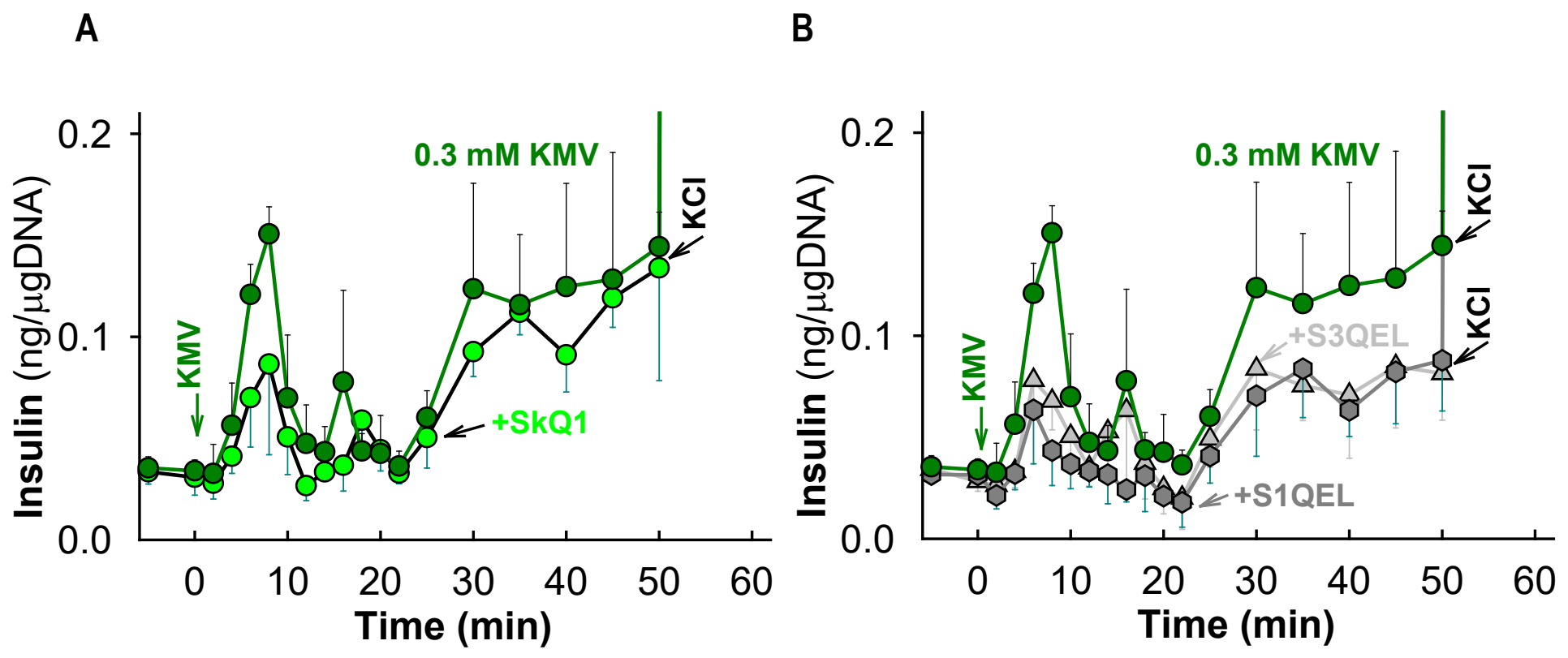

Figure S7. **Insulin secretion rates during PI perifusion with 0.3 mM KMV – A**,**B**) Time course of insulin release rates (amounts released in previous 2 or 5 min) from perifused PIs with 5.5 mM glucose exposed to 0.3 mM KMV (A,B; *dark green*) with SDs of *N*=4 distinct PI isolations. Panel A also includes PI preincubations for 10 min with 10 nM SkQ1 (*green*) and perifusion with the same dose; panels **B** shows preincubations/perifusion with presence of 2 µM S1QEL (*dark gray*) and 2 µM S3QEL (*gray*).

II.8. Elevated glycemia after peritoneal administration of KIC to mice

Our assay of BCKA-SIS in C57Bl6/N mice used intraperitoneal (i.p.) injections of 10 mg KIC per kg and measurements of time courses of blood insulin and glucose in a cohort of mice, similarly, as performed for intralipid [14] and glucose [11]. Three time points were assayed for each individual mouse and resulting data, combined for the entire cohort, were compared with those of iso-osmolar NaCl injections and no treatment (Fig. 7L). Parallel estimations of glycemia are shown in Fig. S8. Evidently, glucose was released from certain organs or tissues, such as liver after injection of either KIC or NaCl. Only a mild glycemia increase was observed when mice were not injected.

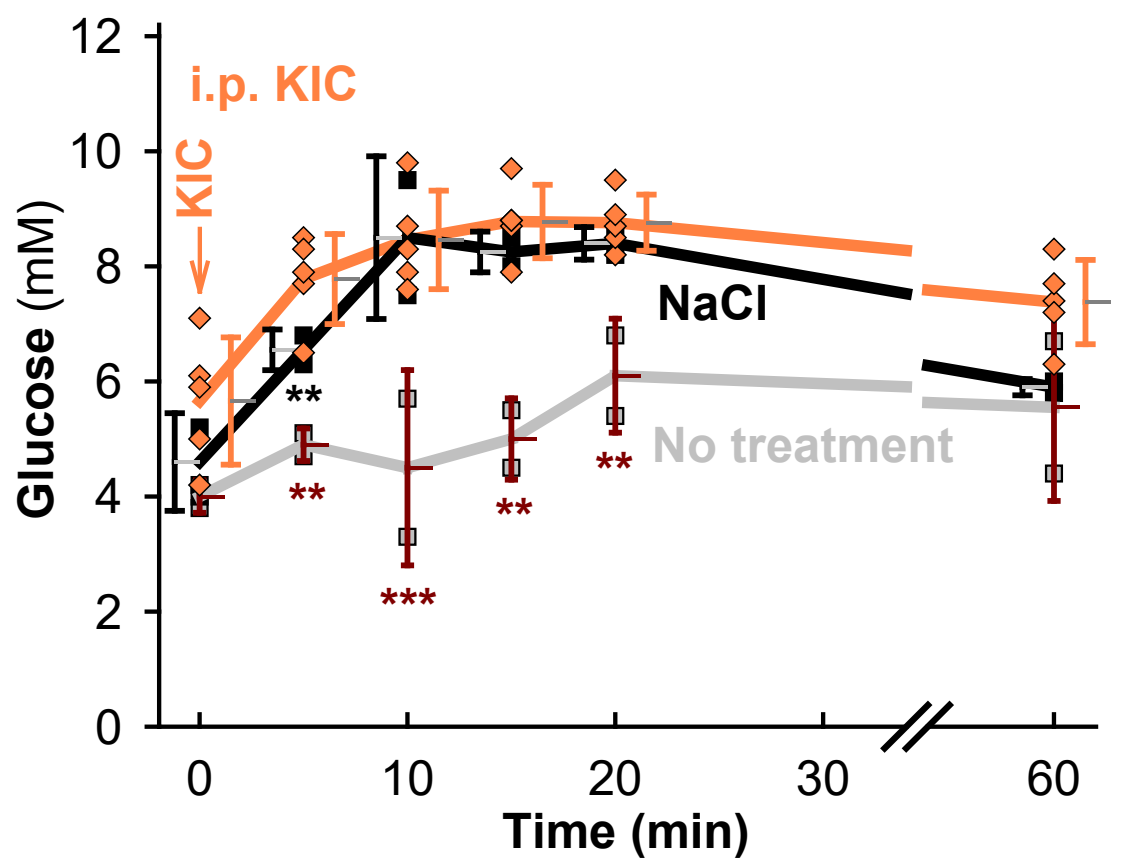

Figure S8. **Glycemia time course after intraperitoneal administration of KIC to mice** – 10 mg per kg of KIC was i.p. injected to mice and glycemia values were recorded three times for each mouse in the cohort. This is compared to i.p. injection of iso-osmolar NaCl and to a cohort of mice which were not injected („No treatment"). T-test: KIC vs. No treatment: ** $P < 0.05$, *** $P < 0.001$; KIC vs. NaCl: ** $P < 0.05$.
